# Phosphorylation of cytochrome *c* at tyrosine 48 finely regulates its binding to the histone chaperone SET/TAF‐Iβ in the nucleus

**DOI:** 10.1002/pro.5213

**Published:** 2024-11-16

**Authors:** Joaquin Tamargo‐Azpilicueta, Miguel Á. Casado‐Combreras, Rafael L. Giner‐Arroyo, Adrián Velázquez‐Campoy, Inmaculada Márquez, José L. Olloqui‐Sariego, Miguel A. De la Rosa, Irene Diaz‐Moreno

**Affiliations:** ^1^ Institute for Chemical Research (IIQ), Scientific Research Center “Isla de la Cartuja” (cicCartuja) University of Seville – CSIC Seville Spain; ^2^ Institute for Biocomputation and Physic of Complex Systems (BIFI), Joint Unit GBsC‐CSIC‐BIFI University of Zaragoza Zaragoza Spain; ^3^ Department of Biochemistry and Molecular and Cellular Biology University of Zaragoza Zaragoza Spain; ^4^ Institute for Health Research Aragón (IIS Aragon) Zaragoza Spain; ^5^ Centre for Biomedical Research Network of Hepatic and Digestive Diseases (CIBERehd) Madrid Spain; ^6^ Department of Physical‐Chemistry University of Seville Seville Spain

**Keywords:** cytochrome *c*, DNA damage response, gene code expansion, histone chaperones, nuclear magnetic resonance, phosphorylation, post‐translational modifications, protein–protein interaction, SET/TAF‐Iβ

## Abstract

Post‐translational modifications (PTMs) of proteins are ubiquitous processes present in all life kingdoms, involved in the regulation of protein stability, subcellular location and activity. In this context, cytochrome *c* (C*c*) is an excellent case study to analyze the structural and functional changes induced by PTMS as C*c* is a small, moonlighting protein playing different roles in different cell compartments at different cell‐cycle stages. C*c* is actually a key component of the mitochondrial electron transport chain (ETC) under homeostatic conditions but is translocated to the cytoplasm and even the nucleus under apoptotic conditions and/or DNA damage. Phosphorylation does specifically alter the C*c* redox activity in the mitochondria and the C*c* non‐redox interaction with apoptosis‐related targets in the cytoplasm. However, little is known on how phosphorylation alters the interaction of C*c* with histone chaperones in the nucleus. Here, we report the effect of C*c* Tyr48 phosphorylation by examining the protein interaction with SET/TAF‐Iβ in the nuclear compartment using a combination of molecular dynamics simulations, biophysical and structural approaches such as isothermal titration calorimetry (ITC) and nuclear magnetic resonance (NMR) and *in cell* proximity ligation assays. From these experiments, we infer that Tyr48 phosphorylation allows a fine‐tuning of the C*c*‐mediated inhibition of SET/TAF‐Iβ histone chaperone activity *in vitro*. Our findings likewise reveal that phosphorylation impacts the nuclear, stress‐responsive functions of C*c*, and provide an experimental framework to explore novel aspects of C*c* post‐translational regulation in the nucleus.

## 1 INTRODUCTION

Tissue homeostasis and normal cell physiology rely on a plethora of interconnected, dynamic mechanisms that are activated in response to endogenous (e.g., oxidative stress, DNA replication stress) and exogenous (e.g., ionizing radiation, oxygen–glucose deprivation) sources of stress (Galluzzi et al. 2018). Most signaling factors participating in these pathways undergo post‐translational modifications (PTMs) that are fundamental to modulate their structure, activity, and localization (Dunphy et al. 2021).

Mitochondria, essential organelles for the production of energy in eukaryotic cells, act as hubs of many of the stress response pathways by (i) producing biosynthetic intermediates and free radicals that act as signaling molecules in the cytosol and nuclei; (ii) sensing and initiating the response to protein misfolding or mistargeting; and (iii) regulating the release of pleiotropic proteins that target the cytoplasm or the nucleus (Bohovych and Khalimonchuk 2016). In this context, cytochrome *c* (C*c*) stands out as a multifunctional factor that (i) acts as a soluble electron carrier in the mitochondrial electron transport chain (ETC) in homeostasis (Alvarez‐Paggi et al. 2017; Díaz‐Quintana et al. 2020; Moore 1990), (ii) is required for apoptosis progression in the cytoplasm (Alvarez‐Paggi et al. 2017; Liu et al. 1996), and (iii) participates as a genotoxic stress response element upon translocation to the cell nucleus under DNA damage (Arif et al. 2016; Nolin et al. 2016; Nur‐E‐Kamal et al. 2004; Xiang et al. 2021). When shuttled to the nucleus, C*c* binds to a number of targets involved in DNA damage response (DDR) that share some structural features although encompassing diverse functions (González‐Arzola et al. 2022; Martínez‐Fábregas et al. 2014a, 2014b). Among them, the oncoprotein SET/template‐activating factor Iβ (SET/TAF‐Iβ) participates in the control of chromatin dynamics as a histone chaperone (Karetsou et al. 2009; Kato et al. 2011; Kawase et al. 1996; Muto et al. 2007; Park et al. 2023; Seo et al. 2001). Furthermore, SET/TAF‐Iβ regulates the activity of protein phosphatase 2A (PP2A), a master regulator of DDR (Di Mambro and Esposito 2022; Li et al. 1996; Ramos et al. 2019) and retains DDR factors at DNA repair foci (Kalousi et al. 2015; Kim et al. 2014). In this context, nuclear translocated C*c* has been reported to hamper SET/TAF‐Iβ binding to histones, thus limiting nucleosome (dis)assembly activity (González‐Arzola et al. 2015) and its modulatory action on PP2A (Casado‐Combreras et al. 2022).

Besides its localization‐dependent function, C*c* can be post‐translationally modified in vivo, both under homeostatic or pathogenic states (García‐Heredia et al. 2012; Guerra‐Castellano et al. 2020; Kalpage et al. 2020). Among the most well‐described PTMs of C*c*, Tyr48 (i.e., the second of the two Tyr residues in the GYSY motif) phosphorylation was first identified in bovine liver tissue (Yu et al. 2008), where reactive oxygen species (ROS) and toxic byproducts are constantly generated as a consequence of this organ's metabolism and detoxifying activity (Conde de la Rosa et al. 2022). Interestingly, a C*c* phosphomimetic mutant generated by substituting Tyr48 by a non‐canonical amino acid (namely, *p‐*carboxymethyl‐L‐phenylalanine, *p*CMF, a phenylalanine residue with a carboxymethyl group bonded to its ζ carbon) through the genetic code expansion technology, was found to have a reduced electron transfer rate and to preclude C*c*‐mediated caspase activation (Gomila et al. 2022; Guerra‐Castellano et al. 2015, 2018; Moreno‐Beltrán et al. 2017). In line with this, other authors have described that Tyr48 phosphorylation has a cytoprotective role, as it reduces the electron flow in the ETC (thus preventing electrons from leaking and generating ROS), while impeding the progression of apoptosis (Pecina et al. 2010).

In this work, isothermal titration calorimetry (ITC) and nuclear magnetic resonance (NMR) experiments with the phosphomimetic Y48*p*CMF mutant allow to conclude that C*c* phosphorylation on Tyr48 leads to (i) a lower binding affinity and a change in the binding mode of C*c* toward SET/TAF‐Iβ; (ii) and, ultimately, to a decreased ability of C*c* to inhibit the histone chaperone activity. Further details on phosphorylation‐induced changes in C*c* structure and subsequent SET/TAF‐Iβ recognition are provided. Immunofluorescence microscopy and proximity ligation assays (PLA) confirm that C*c* phosphomimetic mutants are located at the cell nuclei upon DNA damage response. Based on these findings, we conclude that C*c* Tyr48 phosphorylation might modulate cell tolerance to apoptosis not only by impairing the activation of C*c* pro‐apoptotic targets in the cytoplasm, but also by modulating its binding to the histone chaperone SET/TAF‐Iβ in the nucleus.

## 2 RESULTS

### 2.1 Tyr48 phosphorylation modifies the heme environment by increasing intramolecular cytochrome *c* dynamics

Phosphorylation of Tyr48 changes the affinity of C*c* toward components of the ETC and abolishes apoptosome activation in the cytoplasm. We thus sought to determine if such a PTM also modifies C*c* functioning in the nucleus. Due to strong yield limitations to obtain phosphorylated proteins from tissues and the lack of knowledge about the activity and specificity of kinases catalyzing this modification, we resorted to test three Tyr48 site‐directed substitutions of C*c*: the two classical phosphomimetic substitutions Tyr‐to‐Asp (Y48D) and Tyr‐to‐Glu (Y48E) and the substitution by *p*‐carboxymethyl‐L‐phenylalanine (*p*CMF) non‐canonical amino acid (Y48*p*CMF), which more accurately resembles the volume and hydropathy of the phosphorylated Tyr (Gomila et al. 2022; Guerra‐Castellano et al. 2015; Moreno‐Beltrán et al. 2017; Pérez‐Mejías et al. 2020; Xie et al. 2007).

**FIGURE 1:**
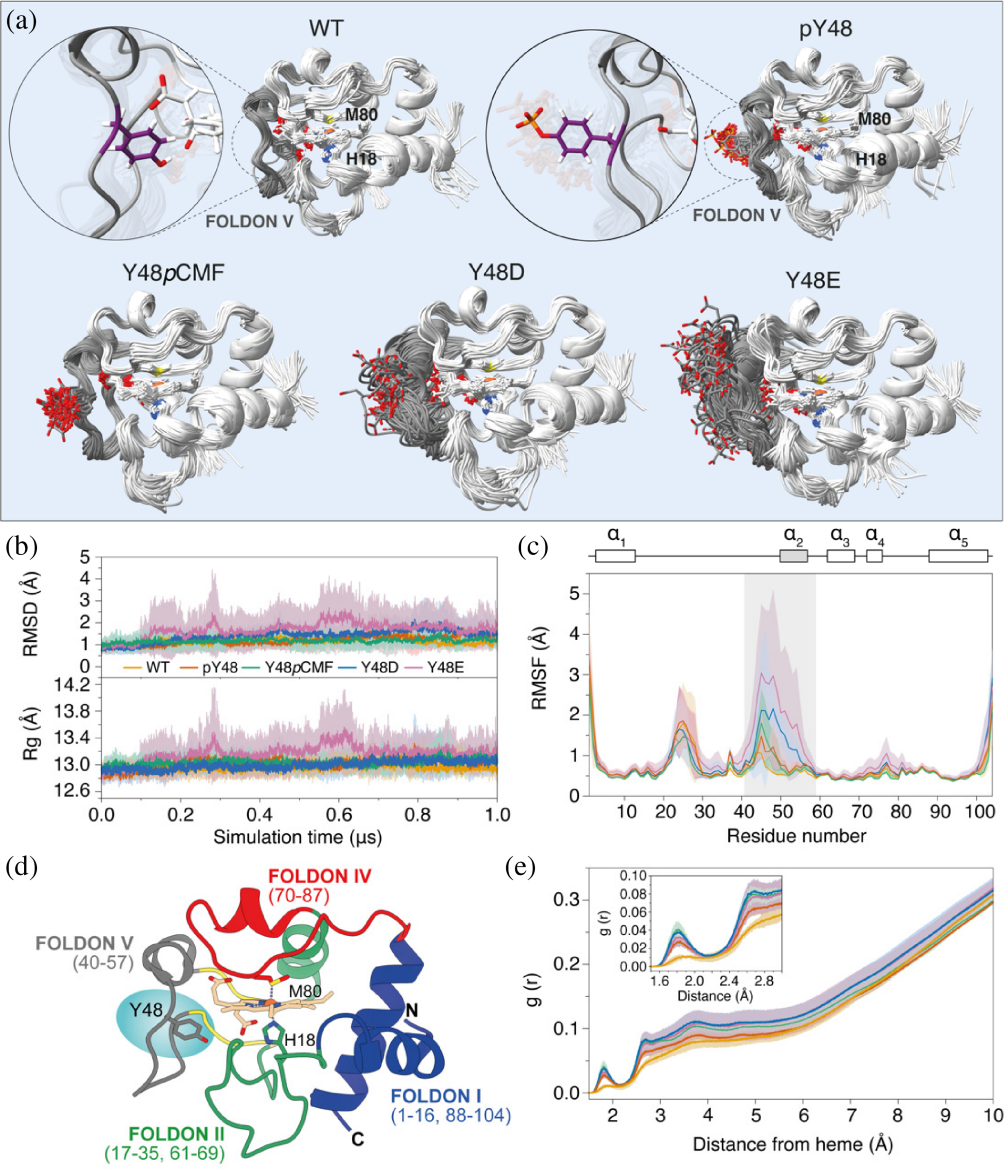
Molecular dynamics simulations of WT, Tyr48‐phosphorylated and phosphomimetic cytochrome *c* species. (a) Overlapping of 10 trajectory snapshots, taken each 100 ns, from representative 1‐μs MD simulations. Tyr48 side chains in unphosphorylated and phosphorylated C*c* are zoomed‐in to illustrate their orientation change induced by phosphorylation. (b) Average backbone atomic root‐mean‐square deviations (RMSD, bottom panel) and radius of gyration (*R*_gyr_, top panel) for the three MD simulation runs. (c) Average atomic root‐mean‐square fluctuations (RMSF) per C*c* residue. Region corresponding to foldon V (residues 40–57), which contains Tyr48, is shadowed in gray. (d) Ribbon representation of C*c* showing its five foldons. Residues involved in each foldon are below the foldon name. (e) Radial distribution function, $g\left(r\right)$, of water molecules around the C*c* porphyrin ring. A spherical region of 10 Å radius around the heme group was analyzed. Average values are represented as thick lines, and standard deviation (±standard error for *n* = 3 replicas) for each replica is represented by a shaded envelope.

To determine the structural resemblance of phosphomimetic Y48*p*CMF, Y48D, and Y48E C*c* species with respect to the Tyr48‐phosphorylated protein, we resorted to molecular dynamics (MDs) simulations. Substitutions by phosphorylated Tyr (pTyr), *p*CMF, Asp, or Glu were introduced to the wild‐type (WT) C*c* NMR‐based model in its reduced state (Imai et al. 2016). Strikingly, whereas the unphosphorylated C*c* Tyr48 sidechain remained facing inwards and contacting with the heme propionate groups, phosphorylation of this residue triggered its rotation toward the solvent (Figure 1a). This reorientation of Tyr48 was also observed in all the phosphomimetic mutants, suggesting that the addition of a negative charge induced by phosphorylation or after its substitution by *p*CMF, Asp or Glu causes its side‐chain exclusion from the heme crevice (Figure 1a).

Radius of gyration (*R*_*g*_) and backbone root‐mean‐square deviation (RMSD) values were stable during all the simulations, whereas slight increases were observed for the Y48E mutant (Figures 1b and S1a,b, Supporting Information). The secondary structure of all the species analyzed remained mostly stable throughout the entire simulation (Figure S2), although the foldon V (residues 40–57) exhibited increased flexibility in the phosphorylated and phosphomimetic species compared to the unmodified WT variant (Figures 1c,d and S1c). Among the phosphomimetics, average RMSF values of the foldon V of the Y48*p*CMF phosphomimetic were comparable to those of the Tyr48‐phosphorylated variant, followed by Y48D C*c*. However, dynamics of the Y48E mutants in this region were markedly enhanced with respect to the phosphorylated variant. For all that, the close proximity (<2.5 Å) of the heme moiety was more accessible for water molecules in the phosphorylated and phosphomimetic variants than in WT C*c* (Figure 1e).

A deeper scrutiny of the distances between the mass centers of the residue at position 48 and the heme iron indicated that this distance is significantly higher in the phosphorylated and phosphomimetic variants than in the unmodified C*c* (Figure S3a,b). Further, the distance RMSD (dRMSD), which provides insights on the changes in the distance of the residue with the rest of the protein, was calculated using the structure at *t* = 0 s as the reference. Interestingly, while the distance and dRMSD values remained stable in the WT, unmodified C*c*, the phosphorylation or phosphomimetic substitution induced a rearrangement in the conformation during the initial steps of the simulation (Figure S3a) that lead to larger distances (around 5 Å further than the WT ones) between the residues at position 48 and the heme iron (Figure S3c).

Similarly to the reduced form, running a 1‐μs simulation of the ferric form of C*c* was enough to reach convergence in RMSD and *R*_*g*_ values (Figure S4a). Likewise, we found that the RMSF values of the foldon V (Figure S4b), the water molecules radial distribution near the heme group (Figure S4c) and the distance of the Tyr48 sidechain to the heme iron (Figure S4d) were enhanced in all the phosphomimetics and in the phosphorylated form compared to the WT, and that the Y48E was the most variable. In fact, secondary structure (Figure S4e) and overall flexibility of the molecule (Figure S4f), was comparable to the reduced form as well.

**FIGURE 2:**
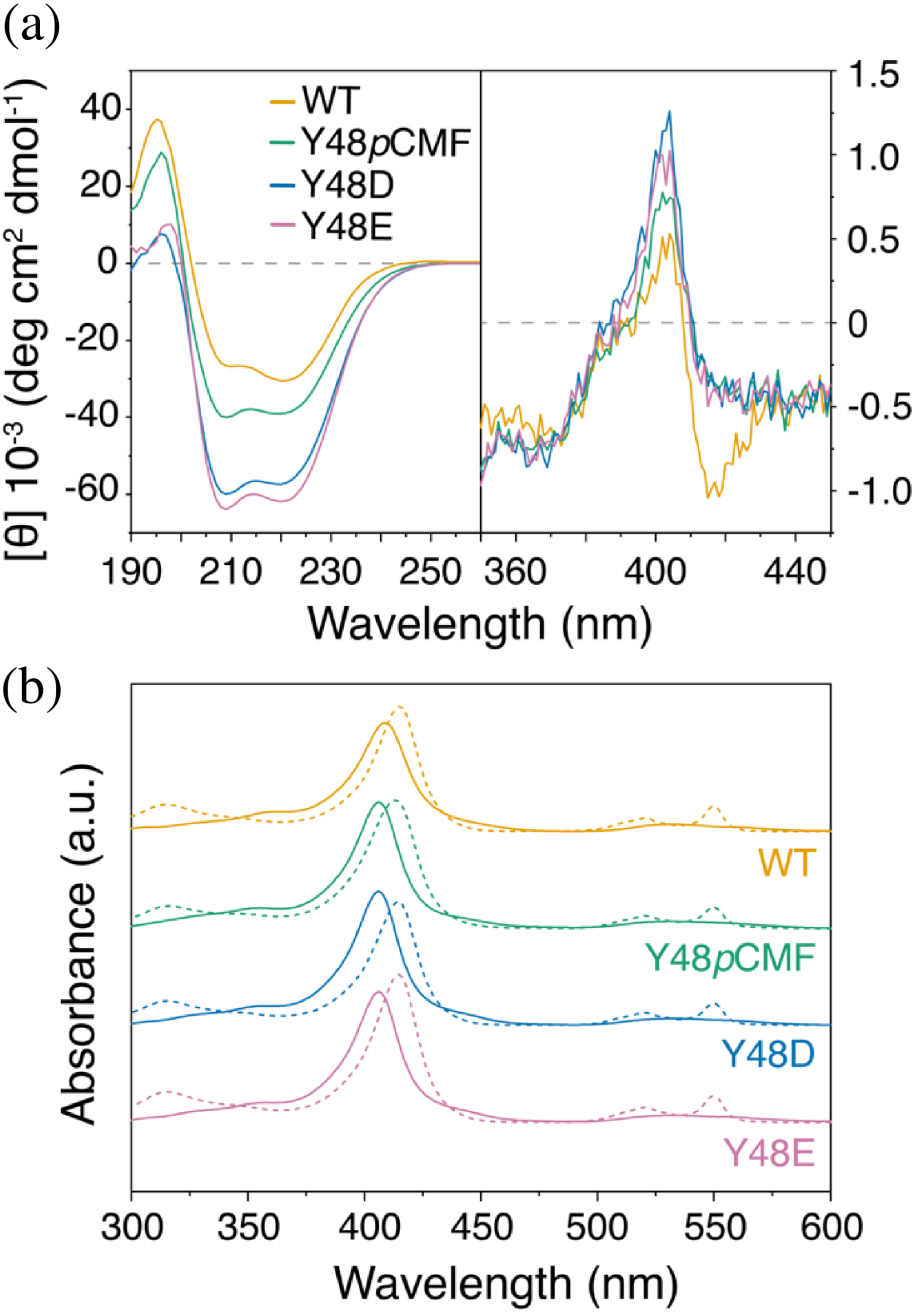
Biophysical characterization of phosphomimetic cytochrome *c* mutants. (a) Far‐UV (left panel) and visible (right panel) CD spectra of oxidized WT and phosphomimetic C*c* mutants Y48*p*CMF, Y48D and Y48E. (b) UV/visible absorption spectra of oxidized (solid lines) and reduced (dashed lines) WT and phosphomimetic C*c* variants. AU, arbitrary units.

Then, Y48*p*CMF, Y48D, and Y48E phosphomimetic mutants were recombinantly expressed to biophysically characterize them and validate the results extracted from MD simulations. Circular dichroism (CD) spectra in the far‐UV region were recorded for all the phosphomimetic mutants in the oxidized state (Figure 2a), showing that the substitution of Tyr48 by *p*CMF, Asp or Glu barely affected the overall secondary structure of C*c* (Table S1), in agreement with MD simulations (Figures S2 and S4e).

At physiological pH values, C*c* heme iron is hexacoordinated with N_δ1_ of His18 and S_δ_ of Met80 as axial ligands. At higher pH values, the S_δ_‐Met80‐coordination of ferric heme iron band is replaced to Lys72, Lys73, or Lys79, in a well‐described process known as alkaline transition (Alvarez‐Paggi et al. 2017; Assfalg et al. 2003; Boffi et al. 2001). Tyr48 substitutions by *p*CMF or Glu are known to disrupt the H‐bond network around the porphyrin ring, thereby facilitating alkaline transition. Indeed, the p*K*_a_ value for alkaline transition has been calculated as 9.3 ± 0.1 for WT C*c* (Guerra‐Castellano et al. 2015), while it decreases to 6.7 ± 0.1 for Y48*p*CMF C*c* (Guerra‐Castellano et al. 2015) and 7.0 ± 0.5 for Y48E C*c* (García‐Heredia et al. 2011). To check if Y48D C*c* follows this behavior, we measured its p*K*_a_ value. A pH titration was performed while recording the charge‐transfer band at 699 nm, which is lost upon alkaline transition (Figure S5) (Schejter and George 1964). In line with the shown decrease in alkaline transition p*K*_a_ by Y48*p*CMF and Y48E phosphomimetic mutants, Y48D C*c* p*K*_a_ shifts to 7.3 ± 0.1. This suggests that intramolecular changes experienced by phosphomimetic C*c* mutants are also reproduced by the Y48D one, in good agreement with the local perturbations predicted by the MD simulations.

To gather information about the heme environment of the phosphomimetic mutants in the oxidized state (Blauer et al. 1993; Santucci and Ascoli 1997), the visible (B‐band, 350–450 nm) CD spectra of WT, Y48*p*CMF, Y48D, and Y48E C*c* were recorded at pH 7.4, which is near to (i) the expected physiological pH of the nucleus and cytoplasm (Casey et al. 2010) and (ii) to the alkaline transition p*K*_a_ values of the phosphomimetic mutants (Figure S5). In fact, while WT C*c* shows a Cotton effect at 410 nm—that is, a positive‐in‐sign and negative‐in‐sign bands typical of the native state in low spin cytochromes (Blauer et al. 1993)—the B‐band splitting is lost in the phosphomimetic mutants (Figure 2a). This splitting is caused by the inner electric field of the protein around the heme porphyrin, and its loss can be attributed to changes in the coordination of the heme iron. Taken together, these results indicate that phosphorylation induces a modification of the heme environment that leads to a drop of the alkaline transition p*K*_a_ to a neutral pH, what highlights a potential biological relevance of the phosphorylation‐induced alkaline transition.

Then, to determine the redox state reversibility of the phosphomimetic mutants, UV/Vis absorption spectra were recorded for WT, Y48*p*CMF, Y48D, and Y48E C*c*, in both their reduced and oxidized state, at pH 7.4 (Figure 2b). All the spectra exhibited the typical hallmarks of *c‐*type cytochromes. In the reduced species, an intense maximum at ca. 415 nm (i.e., Soret band) as well as two lower maximums at 550 and 520 nm (i.e., α and β bands, respectively) were observed. In the oxidized state the α and β bands merged into one single maximum at ca. 530 nm. In all the phosphomimetic species studied, a slight shift on the Soret band maximum was observed in the oxidized state from 409 to 406 nm (Table S2), as previously described in the literature (García‐Heredia et al. 2011). In this context, previous reports pointed out that the Y48*p*CMF mutant exhibited milder differences in redox functionality than the Y48E mutant when compared to WT C*c*, which is in line with the reduced flexibility of foldon V in the former phosphomimetic mutant (Figure 1c) (Guerra‐Castellano et al. 2015; Moreno‐Beltrán et al. 2017). Thus, we attempted to define the magnitude of redox functionality differences between WT and Y48D C*c*. To this end, the thermodynamics and kinetics of the interfacial electron transfer of WT or Y48D C*c* adsorbed onto a gold electrode modified with an 8‐mercaptooctanoic acid (MOA) self‐assembled monolayer (SAM) were determined by performing variable temperature cyclic voltammetry (Figure S6 and Table S3). This monolayer allowed us to simulate the biological protein binding scenario. The standard redox potentials at 25°C associated with the Fe(III)/Fe(II) redox conversion of the immobilized WT and Y48D C*c* heme group were estimated from the midpoint redox potential values (*E*_1/2_) of the raw voltammograms (Figure S6a), resulting in 160 ± 7 mV and 98 ± 5 mV, respectively. Notably, the *E*_1/2_ of immobilized human and horse C*c* at 25°C and pH 7 (Table S3) were substantially lower than the value of ~260 mV vs. NHE determined for both proteins in solution (Oviedo‐Rouco et al. 2022; Rodríguez‐Roldán et al. 2006) due to the relative stabilization of the protein ferric form following its adsorption on a negatively charged thiol monolayer, as observed before for other mammalian, yeast, and bacterial C*c* (de Groot et al. 2007; Kranich et al. 2009; Molinas et al. 2011; Todorovic et al. 2006).

Notably, the Y48D mutant showed a lower redox potential, which is consistent with a higher stabilization of the ferric form upon immobilization. Similar redox potentials were obtained for the previously synthesized Y48*p*CMF and Y48H C*c* phosphomimetic species immobilized under the same binding conditions (Table S3), suggesting that the negative potential shift of Y48D C*c* arise from its higher sensitivity to the electrostatic binding event (Olloqui‐Sariego et al. 2022). The variation of *E*_1/2_ with temperature (Figure S6b) provides the entropic and enthalpic contributions (${∆S}_{\mathrm{rc}}^{0}$ and ${∆H}_{\mathrm{rc}}^{0}$) to the *E*_1/2_ values of the immobilized proteins, by the expression (Battistuzzi et al. 2001; Olloqui‐Sariego et al. 2018, 2020),

(1) $${∆S}_{\mathrm{rc}}^{0}=\mathrm{nF}{\left(\frac{\partial E_{1/2}}{\partial T}\right)}_{P,x_{i}},$$

(2) $${∆H}_{\mathrm{rc}}^{0}=\mathrm{nF}{\left(\frac{\partial \left(E_{1/2}/T\right)}{\partial \left(1/T\right)}\right)}_{P,x_{i}},$$

where *n* = 1, and *F* has its usual meaning. Comparison of the estimated reduction entropies and enthalpies (Table S3) values for both proteins revealed similar ${∆H}_{\mathrm{rc}}^{0}$, and higher ${∆S}_{\mathrm{rc}}^{0}$ absolute values for the Y48D mutant. The lower redox potential of Y48D is thus entropic in origin, suggesting that the tyrosine mutation increases the structure and solvation differences between its oxidized and reduced states.

The effect of the Y48H mutation on the kinetics of the interfacial electron transfer has previously been investigated (Olloqui‐Sariego et al. 2022). For this purpose, the standard electron transfer rate constants (*k*_*s*_) of the proteins were determined from the scan rate dependence of the voltametric peak potentials using the Butler‐Volmer formalism (Laviron 1979), at variable temperature (Figure S6c). Interestingly, the Y48D mutant displayed slightly lower *k*_*s*_ values at lower temperatures, but became higher at increasing temperatures, thereby suggesting different activation parameters to the interfacial electron transfer rate between WT and Y48D C*c*. These activation parameters, the pre‐exponential factors (A) and activation enthalpies (${∆H}_{\mathrm{ET}}^{\#}$) associated with *k*_*s*_ can be obtained from the corresponding Arrhenius plot (Figure S6b) according to

(3) $$\ln\;k_{s}=\ln\;A-\frac{{∆H}_{\mathrm{ET}}^{\#}}{\mathrm{RT}},$$

where the intercept and slope of these plots provide the A and ${∆H}_{\mathrm{ET}}^{\#}$ values, respectively, which are collected in Table S3. Interestingly, the tyrosine mutation provokes a significant increase in both the pre‐exponential factor and the activation enthalpy. This switch of activation parameters was similar to those reported for other Tyr48 mutants (Olloqui‐Sariego et al. 2022), suggesting that such a change is largely independent of tyrosine mutation. The orientation of the adsorbed C*c* onto SAM carboxylated organic surfaces is ruled by interactions with Lys residues 13, 72, 73, 79, 86, 87, and 88 (Xu and Bowden 2006), which are predicted to be unaffected by the mutation (Figures S4f and S6d). Therefore, the substantial rise in the pre‐exponential factor can arise from a structural protein rearrangement that brings the heme ring closer to the electrode. Further, the two‐fold higher activation enthalpy of Y48D compared to that of WT C*c* points toward a greater contribution of the solvent in the activation process. These results are in agreement with the higher mobility of foldon V in the Y48D mutant inferred from MDs, enhancing the accessibility of water molecules to the heme moiety and enabling a large approach between the heme crevice and the electrode.

Altogether, these findings suggest that the redox state of phosphomimetic C*c* retains its reversibility, although the porphyrin environment and its solvent accessibility were perturbed. Furthermore, our observations indicate that the Y48D C*c* phosphomimetic mutant is closer to replicate the structural and biophysical characteristics of the Tyr48‐phosphorylated and Y48*p*CMF phosphomimetic species than the Y48E phosphomimetic variant.

### 2.2 Tyr48 phosphorylation weakens and modifies the binding mode of cytochrome *c* to SET/TAF‐Iβ

In order to check whether Tyr48 phosphorylation changes the C*c* activity in the nucleus, as is the case at the mitochondrial (Gomila et al. 2022; Guerra‐Castellano et al. 2018; Lee et al. 2006; Moreno‐Beltrán et al. 2017; Yu et al. 2008) and cytoplasmic (García‐Heredia et al. 2011; Moreno‐Beltrán et al. 2017; Pecina et al. 2010) spaces, we analyzed the phosphorylation effect on the binding of C*c* to SET/TAF‐Iβ, a nuclear histone chaperone involved in DDR at multiple levels (Kalousi et al. 2015; Karetsou et al. 2009; Kato et al. 2011; Li et al. 1996). The interface used by C*c* to recognize SET/TAF‐Iβ encompasses residues around the heme crevice, including many residues from foldon V (i.e., Ser47, Tyr48, Ala50, Ala51, Lys53, Gln54, Lys55, Gly56) and the 20–35 loop (i.e., Val20, Lys22, Gly23, Gly24, Lys25, Gln31, His33, Leu35) (Casado‐Combreras et al. 2022, 2024; González‐Arzola et al. 2015), which were predicted to have an enhanced flexibility within the Tyr48‐phosphorylated and phosphomimetic species Y48*p*CMF, Y48D, and Y48E (Figure 1).

ITC experiments showed that the interaction between reduced WT C*c* and SET/TAF‐Iβ is entropically driven (binding enthalpy, Δ*H* = 1.3 kcal mol^−1^; entropic term, –*T*Δ*S* = −8.8 kcal mol^−1^) with a dissociation constant (*K*_D_) of 3.4 μM, a C*c*:SET/TAF‐Iβ stoichiometry of 2:1, and a binding mode with modest positive cooperativity at 25°C; in fact, the affinity coupling between the two sites is quite small (ρ = 1.1, ∆g = 0.1 kcal mol^−1^), but the enthalpic coupling is considerable, reflected in a biphasic profile in the binding isotherm (Figure S7 and Table 1), as previously reported (González‐Arzola et al. 2015). Both binding events are entropically driven, but the second one shows a much larger entropic contribution and a more unfavorable enthalpy. Remarkably, all C*c* phosphomimetic mutants exhibited a complete loss of the cooperative binding to SET/TAF‐Iβ, as inferred from the monophasic profile of binding isotherms (Figure S7), although the C*c*:SET/TAF‐Iβ binding stoichiometry was conserved, remaining close to 2:1 (Figure S7 and Table 1). Additionally, there was a notable decrease in the binding affinity by 7‐fold with Y48*p*CMF and Y48D C*c*, and by 30‐fold with Y48E C*c* (Table 1). Moreover, the binding of Y48*p*CMF, Y48D, and Y48E C*c* to SET/TAF‐Iβ became enthalpically driven (∆H < −4.6 kcal mol^−1^; −T∆S = 13.6 kcal mol^−1^). This is consistent with a strong disruption of the electrostatics between the highly acidic surface of SET/TAF‐Iβ and the positively charged heme surroundings of WT C*c*—as reflected by the entropically driven binding mode (Cooper 2005)—induced by the additional negative charge in the phosphomimetic species. It is noteworthy that the decrease in the affinity for SET/TAF‐Iβ exhibited by C*c* mutants (Table 1) is proportional to the increase in mobility experienced at foldon V, likely penalizing the affinity through a larger conformational entropic loss (Figure 1). Taken together, these results suggest a contribution of phosphorylation‐induced intramolecular dynamics that could explain the observed binding changes. In line with this finding, the Y48E mutant showed the most different thermodynamic profile and highest structural distortions, as previously mentioned (Figure 1), as well as a significant increase in *K*_D_ (Table 1). In summary, these findings point out that Tyr48 phosphorylation—when it is mimicked by *p*CMF, Asp, or Glu—causes a drastic effect on the binding of C*c* toward SET/TAF‐Iβ.

**TABLE 1:** Thermodynamic parameters for SET/TAF‐Iβ complexes with reduced WT and mutant cytochrome *c* species.

Protein complex	*K*_D1_ (μM)	Δ*G* (kcal mol^−1^)	Δ*H*_1_ (kcal mol^−1^)	−*T*Δ*S*_1_ (kcal mol^−1^)	*K*_D2_ (μM)	Δ*H*_2_ (kcal mol^−1^)	−*T*Δ*S*_2_ (kcal mol^−1^)	*n*
WT C*c*:SET/TAF‐Iβ	3.4	−7.5	1.3	−8.8	3.1	6.9	−14.4	1.2
Y48*p*CMF C*c*:SET/TAF‐Iβ	23	−6.3	−4.6	−1.7	‐	‐	‐	2.2*
Y48D C*c*:SET/TAF‐Iβ	22	−6.3	−7.5	1.2	‐	‐	‐	2.2*
Y48E C*c*:SET/TAF‐Iβ	110	−5.4	−19.0	13.6	‐	‐	‐	2.3*

*Note*: Calculated equilibrium dissociation constant (*K*_D_), Gibbs free energy (Δ*G*), binding enthalpy (Δ*H*), entropic term (−*T*Δ*S*). *n* stands for (i) the protein active fraction in the WT C*c*:SET/TAF‐Iβ complex fitted to a two‐binding‐sites binding model, or (ii) the binding stoichiometry of complexes involving C*c* phosphomimetic mutants with independent binding sites, indicated using an asterisk (*).

### 2.3 Tyr48‐phosphorylated cytochrome *c* is less capable of inhibiting SET/TAF‐Iβ histone chaperone activity

**FIGURE 3:**
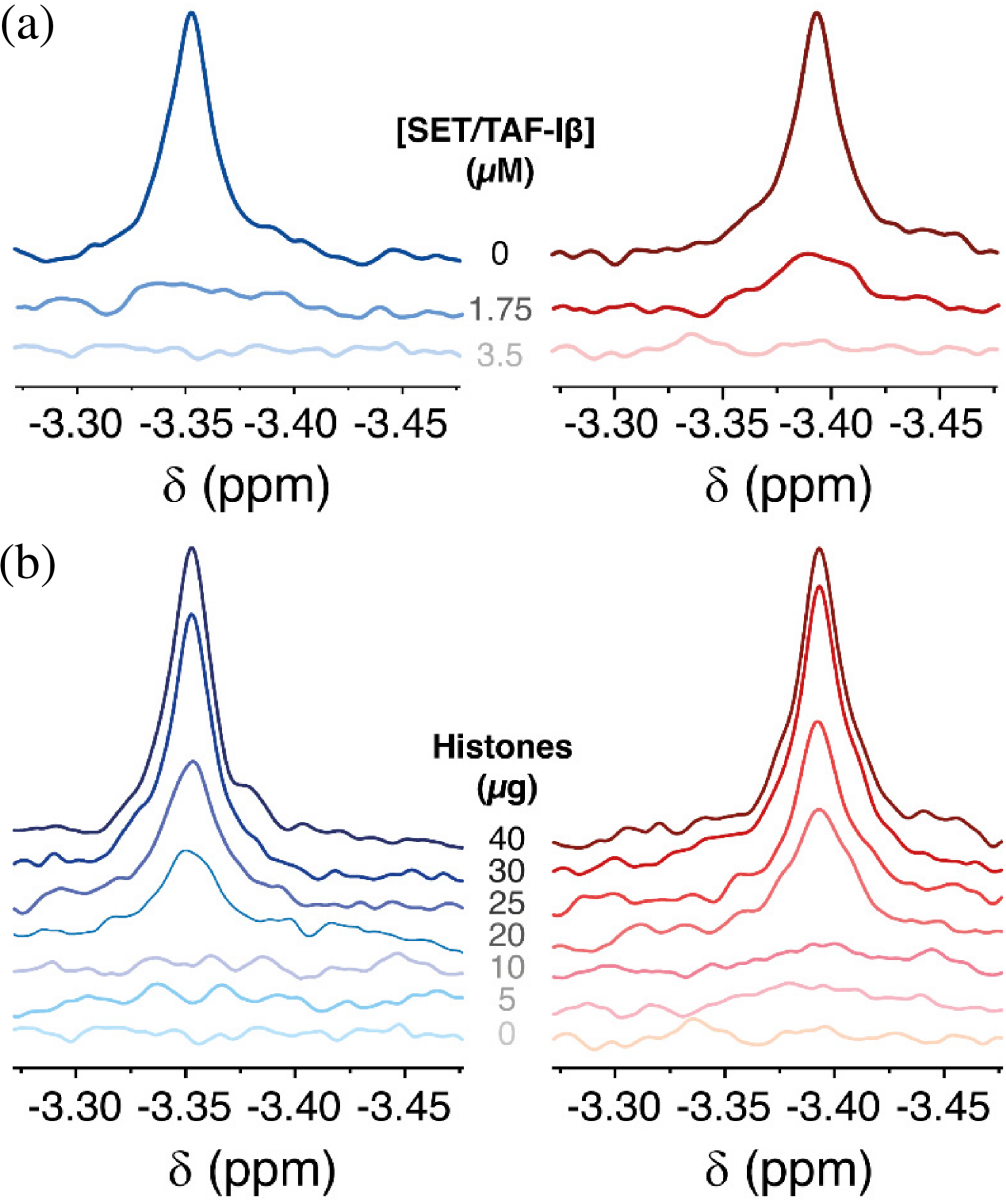
NMR titration of WT and phosphomimetic cytochrome *c* species with SET/TAF‐Iβ and histones. (a) 1D ^1^H NMR spectra monitoring the Met80‐εCH_3_ signal of reduced WT (left) or Y48*p*CMF (right) C*c* (13 μM) in free state and in the presence of increasing concentrations of SET/TAF‐Iβ (0 μM, C*c*:SET/TAF‐Iβ ratio 1:0; 1.75 μM, C*c*:SET/TAF‐Iβ ratio 1:0.14; 3.5 μM, ratio 1:0.27). (b) 1D ^1^H NMR spectra monitoring the WT (left) and Y48*p*CMF (right) C*c* Met80‐εCH_3_ signal in the presence of saturating concentration of SET/TAF‐Iβ and upon addition of increasing amounts of calf thymus histones.

To test whether the changes in structure and binding affinity of C*c* toward SET/TAF‐Iβ by Tyr48 phosphorylation affected its nuclear function, we performed a competition assay recording 1D ^1^H NMR spectra of reduced C*c* in the presence of SET/TAF‐Iβ and increasing concentrations of a mixture of histones (Figure 3).

First, the differences in binding of WT and Y48*p*CMF C*c* to SET/TAF‐Iβ were confirmed by monitoring the broadening of the Met80‐εCH_3_ NMR signal of the heme Fe(II) axial ligand at different SET/TAF‐Iβ:C*c* ratios (Figures 3a and S8a). The addition of SET/TAF‐Iβ to a C*c* sample at a 1:0.14 molar ratio (C*c*:SET/TAF‐Iβ) yielded a complete broadening of the Met80‐εCH_3_ signal beyond the detection limit with WT C*c*, but not with the Y48*p*CMF species. The prominent signal in the Y48*p*CMF C*c* spectra at the indicated ratio denotes fast exchanging populations between the unbound and bound states of Y48*p*CMF C*c*. On the other hand, WT C*c* signal remained at the baseline level at this ratio, suggesting that WT C*c* is mostly bound. Increasing the concentration of SET/TAF‐Iβ up to a 1:0.27 ratio (C*c*:SET/TAF‐Iβ) yielded a total broadening of the Met80‐εCH_3_ signal with both WT and Y48*p*CMF species. These results are consistent with the calorimetry data, which indicated a lower binding affinity of the phosphomimetic C*c* toward SET/TAF‐Iβ than the unmodified, WT C*c* (Table 1). As a control of binding specificity, the linewidth of the C*c* Met80‐εCH_3_ signal was monitored and found to be unaffected upon addition of BSA (Figure S8b).

To corroborate that the phosphomimetic species of C*c* retained the ability to inhibit SET/TAF‐Iβ histone chaperone activity, we measured the recovery of the Met80‐εCH_3_ signal upon addition of increasing histones amounts (Figure 3b). The increase in the signal intensity served as an indicator of C*c* release from its complex with SET/TAF‐Iβ, hence revealing a competition between the hemeprotein and histones. Both WT and Y48*p*CMF C*c* are completely displaced from the complex with SET/TAF‐Iβ when adding 40 μg of a calf thymus histones mixture. Notably, the Met80‐εCH_3_ signal intensity recovery is gradual upon adding increasing amounts of histones, indicating that Y48*p*CMF C*c* still retain the ability to compete with histones for SET/TAF‐Iβ binding sites. According to the fitting of the normalized maximum intensity of the Met80‐εCH_3_ NMR signal, the histone/C*c* (His/C*c*) mass ratio required to inhibit the histone chaperone activity of SET/TAF‐Iβ to a half (i.e., the half maximal inhibition concentration, EC50), was slightly reduced from 0.77 (±0.026) for WT to 0.69 (±0.046) for Y48*p*CMF C*c* (Figure S8c). Altogether, these results indicate that phosphorylation does not fully impair the C*c*‐mediated inhibition of SET/TAF‐Iβ histone chaperone activity but modulates the hemeprotein binding to SET/TAF‐Iβ and the ability to compete against histones (Figure 3b).

### 2.4 Tyr48‐phosphomimetic cytochrome *c* is able to shuttle to the cell nucleus under basal DNA damage and interact with SET/TAF‐Iβ

Tyr48 phosphomimetic C*c* variants undergo structural and dynamical modifications that affect their recognition of SET/TAF‐Iβ, as reflected in their mode of competition with histones for binding sites on the chaperone. We then wanted to test whether phosphomimetic C*c* species are also able to translocate to the nucleus and recognize SET/TAF‐Iβ *in cell*.

**FIGURE 4:**
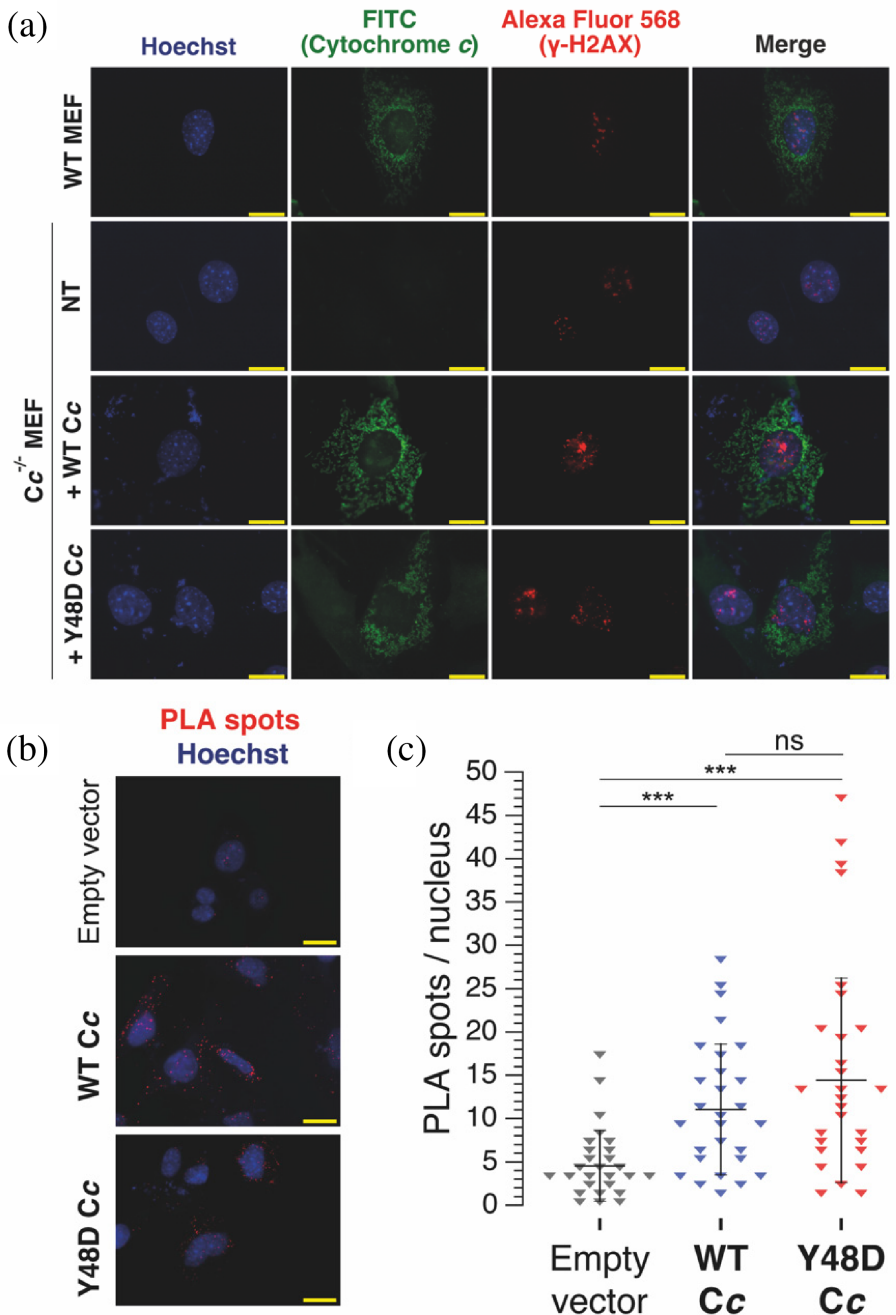
Interaction of WT and Y48D cytochrome *c* with SET/TAF‐Iβ in the nucleus of MEF cells on basal DNA damage levels. (a) Immunofluorescence analyses of γ‐H2AX and C*c* in WT (endogenous C*c*) and C*c* ^−/−^ (transfected or not with WT or Y48D C*c* species) MEF cells. The subcellular distribution of C*c* and γ‐H2AX was visualized using anti‐C*c* and anti‐γ‐H2AX antibodies—namely, FITC and Alexa Fluor 568, respectively. NT stands for non‐transfected cells but treated equally to the transfected and WT control cells, including incubation with both primary and secondary antibodies. Scale bars are 20 μm. (b) In situ PLA detection of C*c*‐SET/TAF‐Iβ complexes and immunofluorescence labeling of C*c* in C*c* ^−/−^ MEF transfected with an empty vector, WT C*c* or Y48D C*c*. Representative projections of each condition with PLA spots in red and nuclei in blue are shown. Scale bars are 20 μm. (c) Quantitation of PLA spots per nucleus (*n* = 27 for empty vector, *n* = 28 for WT C*c*, and *n* = 27 for Y48D C*c*). Black lines and whiskers represent the mean and the SD, respectively. Statistical significance was calculated using the Mann–Whitney test (***p < 0.001; “ns” stands for nonsignificant).

First, to test whether phosphomimetic C*c* species can redistribute to the nucleus, we monitored the C*c* subcellular localization by immunofluorescence in mouse embryonic fibroblasts (MEFs) deprived of both copies of the C*c* genes (C*c* ^−/−^) and transfected with constructs encoding WT or phosphomimetic Y48D human C*c*. Notably, we found that both WT and C*c* ^−/−^ MEF cells showed basal formation of γ‐H2AX foci, an indicator of the activation of the DNA damage response (DDR; Mah et al. 2010; Paull et al. 2000). Basal DNA damage under homeostatic conditions with subsequent formation of DNA foci has been observed for other cell lines (Guard et al. 2019). In consequence, endogenous C*c* was mainly localized in mitochondria of WT MEFs but was also detected in the nuclei that contained γ‐H2AX foci (Figure 4a). As a control, no fluorescence was observed when using the polyclonal anti‐C*c* antibody in C*c* ^−/−^ MEF cells when transfected with an empty vector.

The Y48D phosphomimetic variant was chosen for *in cell* experiments instead of the Y48*p*CMF mutant, as the introduction of *p*CMF species in eukaryotes using the gene code expansion remains challenging in hemeproteins because of their particular folding route that involves entering in the mitochondria (Dumont et al. 1988, 1991; Grasso et al. 2022; He et al. 2022; Niu and Guo 2023). From our observations herein reported, Y48D C*c* is structurally and biophysically closer to pY48 and Y48*p*CMF C*c* than the Y48E mutant (Figures 1 and S1–S4) and further reproduces the Y48*p*CMF interplay with SET/TAF‐Iβ (Table 1).

C*c* ^−/−^ MEFs transfected with WT or Y48D C*c* expression vectors also exhibited γ‐H2AX foci formation, as did WT MEFs. They also showed a mainly mitochondrial localization of C*c*, as well as a nuclear pool of unmodified or phosphomimetic C*c*.

In this framework, we wanted to determine if the Y48D C*c* phosphomimetic was also able to physically interact with endogenous SET/TAF‐Iβ *in cell*. To this end, PLA was performed in these cells (Figure 4b,c). Following transfection, PLA spots were detected in the nucleus, evidencing interaction between SET/TAF‐Iβ and both variants of the heme protein. No significant differences were found between the number of nuclear PLA spots of WT and Y48D C*c*, which could be ascribed to changes in transfection or protein expression levels, among others. The interaction between SET/TAF‐Iβ and C*c* was also widely detected in the cytosol. This interaction may take place following the cytosolic localization of SET/TAF‐Iβ upon being post‐translationally modified (Nagata et al. 1998; Vera et al. 2007; Yu et al. 2013), if there is an available C*c* cytosolic pool originating from the mitochondrial release. Altogether, these results show that a pool of phosphomimetic C*c* reaches the nucleus of cells under active DDR, and that such a post‐translationally modified C*c* population are still able to interact with SET/TAF‐Iβ.

## 3 DISCUSSION

As a pleiotropic regulator of cell life and death, C*c* is comprehensively subjected to PTMs that affect its redox activity in the mitochondria and pro‐apoptotic function in the cytosol (García‐Heredia et al. 2011; Gomila et al. 2022; Guerra‐Castellano et al. 2020; Kalpage et al. 2020; Moreno‐Beltrán et al. 2015, 2017; Olloqui‐Sariego et al. 2022; Pérez‐Mejías et al. 2020). In this work, we broaden the functional scope of PTMs over C*c* to the cell nucleus, where the hemeprotein is translocated under genotoxic stress (Alvarez‐Paggi et al. 2017; Arif et al. 2016; Casado‐Combreras et al. 2022; González‐Arzola et al. 2015, 2022; Martínez‐Fábregas et al. 2014a, 2014b; Moreno‐Beltrán et al. 2015; Nolin et al. 2016; Nur‐E‐Kamal et al. 2004; Rivero‐Rodríguez et al. 2021; Xiang et al. 2021; Zhou et al. 2024), demonstrating that Tyr48 phosphorylation modifies its binding toward its nuclear partner SET/TAF‐Iβ in vitro.

Tyr48, along with Gly41, Gly45, Ala51, Asn42, and Lys55, are the most evolutionary conserved residues of the C*c* foldon V (residues 40–57; Figure S9), which is the C*c* substructure with the lowest unfolding free energy. Interestingly, four out of the six described pathogenic, naturally occurring C*c* missense mutations (i.e., G41S, Y48H, A51V, A51T) fall within this foldon (De Rocco et al. 2014; Marzollo et al. 2024; Morison et al. 2008; Ong et al. 2017). Our MD simulations suggest that the sidechains of phosphorylated Tyr and phosphomimetic *p*CMF at position 48 are excluded from the heme environment and turn outwards, enlarging the heme crevice and thus altering the alkaline transition and redox properties of the protein. This is consistent with marked differences in the orientation of Tyr48 sidechain and the local structure of foldon V between NMR‐resolved structures of WT (PDB ID: 2N9I; Imai et al. 2016) and Y48*p*CMF (2N3Y; Moreno‐Beltrán et al. 2017). Likewise, the Y48H thrombocytopenia‐related C*c* mutant was found to have increased dynamics in the 40–57 and 71–85 loops (Deacon et al. 2017), along with a drop in the alkaline transition p*K*_a_ (Deacon et al. 2018; Deng et al. 2019). These findings indicate that Tyr48 controls the rigidity of the foldon V and, consequently, the alkaline transition. However, unlike the Y48H missense mutation, PTMs are reversible, allowing cells to switch between two active configurations of C*c* based on the cellular status.

Tyr48 phosphorylation plays a cytoprotective role by decelerating the electron flux in mitochondrial ETC (Gomila et al. 2022; Pecina et al. 2010; Yu et al. 2008), decreasing ROS production (Moreno‐Beltrán et al. 2017; Pecina et al. 2010), and impeding caspase activation (Moreno‐Beltrán et al. 2017; Pecina et al. 2010). Here, we show that Tyr48 phosphorylation further modulates the binding to one of its nuclear partners (namely, the histone chaperone SET/TAF‐Iβ). ITC experiments revealed a ca. 10‐fold reduction in the binding affinity of Y48*p*CMF and Y48D C*c* phosphomimetic mutants toward SET/TAF‐Iβ (Figure 3 and Table 1). These results suggest that the increase in foldon‐V mobility and the addition of a negative phosphate charge—mimicked by *p*CMF, Asp or Glu mutations—hampers the binding to SET/TAF‐Iβ, by altering orientation of residues important for the chaperone recognition and distorting the charge complementarity. This leads to a shift of the thermodynamic binding profile from an entropically driven binding to an enthalpically driven binding and to the loss of binding cooperativity (Table 1).

Despite reduced binding, 1D ^1^H NMR experiments of WT and Y48*p*CMF C*c* show that the phosphomimetic C*c* still retains its ability to compete with histones for binding sites in SET/TAF‐Iβ, albeit to a mildly lesser extent than the WT protein. Consistent with the cytoprotective role of C*c* Tyr48 phosphorylation in the cytoplasm, nuclear‐translocated phosphorylated C*c* would be partially impeded to inhibit SET/TAF‐Iβ chromatin remodeling activity, thus fostering the access of DDR factors to DNA damage foci in cell tissues persistently exposed to endogenous genotoxic stress. In cell, PLA experiments evidenced that the C*c* phosphomimetic variant Y48D could access the cell nucleus and bind SET/TAF‐Iβ (Figure 4). While the number of PLA spots was comparable in both WT and Y48D C*c*, different cytosol/nuclear shuttling rates or retention by other nuclear partners, among several other factors, might occur. Future studies should address the C*c* nuclear import pathway, how it can be modulated by the phosphorylation and whether the change in binding dynamics toward SET/TAF‐Iβ upon phosphorylation effectively results in a change in the chromatin remodeling or other activities of the histone chaperone.

In summary, the results herein reported shed light on the mechanism by which phosphorylation of C*c* Tyr48 finely tunes the binding to SET/TAF‐Iβ and, consequently, its histone chaperone activity. In this context, a possible effect of Tyr48 phosphorylation on other C*c* functionalities exerted through its binding to SET/TAF‐Iβ (as a histone chaperone or as a modulator of PP2A activity, among others), or other nuclear partners like nucleophosmin (NPM1) or acidic leucine‐rich nuclear phosphoprotein 32 family member B (ANP32B), cannot be excluded. Further, these results invite to scrutiny unexplored facets of other in vivo identified PTMs of C*c* (e.g., nitration, acetylation, methylation).

Collectively, our findings add up to the decreased electron transfer rate of C*c* at the ETC and the lower caspase activation ability observed for Y48*p*CMF C*c* in its mitochondrial and cytoplasmic roles, respectively. Phosphorylation of Tyr48 causes significant changes in structure dynamics that impact the binding to SET/TAF‐Iβ and lessen the C*c*‐mediated inhibition of SET/TAF‐Iβ histone chaperone activity. Altogether, these findings reveal that phosphorylation impact the nuclear, stress‐responsive functions of C*c* and broaden the scope of the impact of C*c* posttranslational regulation in the nucleus.

## 4 MATERIALS AND METHODS

### 4.1 Molecular dynamics simulations

The initial C*c* structures for molecular dynamics (MD) calculations were built based on the NMR structure of human C*c* in its reduced state (PDB ID 2N9I; Imai et al. 2016). Structural models of Tyr48‐phosphorylated (in a monoprotonated state) and phosphomimetic C*c* mutants (Y48*p*CMF, Y48D, and Y48E) were built using the WT, unphosphorylated protein as a template. The force field parameters of monoprotonated phosphotyrosine (Homeyer et al. 2006), *p*CMF (Guerra‐Castellano et al. 2015), and Fe(II/III)‐heme group (Autenrieth et al. 2004) were used to obtain the topology and coordinate files required for the simulation using the TLEaP module of AMBER20 (Case et al. 2020).

MD simulations were carried out using the OpenMM toolkit, version 7.4.2 (Eastman et al. 2017) and the AMBER‐14SB force‐field libraries (Maier et al. 2015). Simulations were performed under periodic boundary conditions, using an orthorhombic cell geometry and particle mesh Ewald (PME) electrostatics, with an Ewald summation cut off of 8 Å. The system was neutralized with sodium and chlorine counter‐ions according with the total charge of the protein and solvated using an optimal 3‐charge, 4‐point rigid water model (OPC) molecules (Izadi et al. 2014). The whole system was subjected to 2500 steps of energy minimization at 298 K. Temperature was regulated by using a Langevin thermostat (Andersen 1980) with a friction coefficient of 1 ps^−1^ and a step size of 0.002 ps. For each C*c* species, three replicas beginning at different coordinates were subjected to 1 μs of MD simulation.

CPPTRAJ module of AMBER was used for the trajectory analysis (De la Cerda et al. 1997; Roe and Cheatham 2013; Romero et al. 1998). OriginPro 2024b was used for plotting and statistical analysis. Molecular graphics were depicted with UCSF ChimeraX 1.8 software (Meng et al. 2023).

### 4.2 DNA constructs

For cellular localization experiments, the gene encoding WT or Y48D C*c* were cloned into the pcDNA3.1(+) vector, alongside with a C‐terminal *c*‐myc tag (aminoacidic sequence: EQKLISEEDL). Cloning was achieved using BamHI and XbaI restriction enzymes (New England Biolabs). DNA inserts for cloning were obtained via PCR from cDNA gBlocks purchased from Genecust (France), using the following primers: 5′‐TAGCTTGGTACCGAGCTCGGATCCATGGGCGACGTGGAAAAGGGCAAAAAG‐3′ and 5′‐GGGCCCTCTAGACTCGAGCTACAGATCCTCTTCAGAGATGAGTTTCTGCT‐3′.

For the recombinant expression of SET/TAF‐Iβ, the pET28a(+) bacterial expression vector containing the gene encoding the protein fused to a N‐terminal 6xHis‐tag was used (González‐Arzola et al. 2015).

For the recombinant expression of WT C*c* and its Y48D and Y48E phosphomimetic C*c* mutants, constructs were cloned in a pBTR1 vector which also contained the *CYC3* gene, encoding the yeast heme lyase required for the proper folding of C*c* when expressed in bacterial cell cultures (Moreno‐Beltrán et al. 2015; Olteanu et al. 2003). The plasmids for Y48D and Y48E C*c* expression were obtained by site‐directed mutagenesis of pBTR1, using the following PCR primers: 5′‐GATACGGCGGCGAACAAAAAC‐3′ and 5′‐ATCGCTGTAGCCCGGCGCCTG‐3′ (Y48D) or 5′‐GAAACGGCGGCGAACAAAAAC‐3′ and 5′‐TTCGCTGTAGCCCGGCGCCTG‐3′ (Y48E).

For the recombinant expression of the Y48*p*CMF phosphomimetic mutant, two bacterial expression plasmids were required: (i) a pBTR1 vector encoding a C*c* sequence where Tyr48 codon was substituted to an Amber stop codon (TAG) and (ii) a pEVOL vector, that encodes an unnatural tRNA/aminoacyl‐tRNA synthetase (uaaRS) pair specific for the introduction of *p*CMF into the TAG codon (Guerra‐Castellano et al. 2015).

All constructs were confirmed through automated sequencing. All oligonucleotides were purchased from Sigma.

### 4.3 Protein expression and purification

The recombinant expression of SET/TAF‐Iβ was performed as previously described (González‐Arzola et al. 2015). Single clones of *Escherichia coli* BL21(DE3) cells transformed with the expression plasmid and inoculated into 2.5 L of LB medium supplemented with 50 μg mL^−1^ kanamycin. Cultures were grown up to OD_600_ = 0.8 at 37°C under agitation (180 rpm) and then, induced with 1 mM isopropyl‐β‐D‐1‐thiogalactopyranoside (IPTG) and incubated under agitation (180 rpm) at 30°C overnight.

WT, Y48D, and Y48E C*c* were expressed as previously described (Gomila et al. 2022; Guerra‐Castellano et al. 2015; Moreno‐Beltrán et al. 2015). Protein expression was induced by culturing at 30°C with agitation (180 rpm) in LB supplemented with 100 μg mL^−1^ ampicillin for 20 h.

For the expression of Y48*p*CMF C*c* mutants, *E. coli* BL21(DE3) cells were co‐transformed with pEVOL:uaaRS and pBTR1:Y48AMBER vectors (Guerra‐Castellano et al. 2015; Moreno‐Beltrán et al. 2017). Selected clones were cultured at 37°C and 180 rpm in M9 minimal medium, supplemented with 100 μg mL^−1^ ampicillin and 20 μg mL^−1^ chloramphenicol, until they reached OD_600_ = 0.6. Then, cultures were treated with 0.02% arabinose and 1 mM IPTG to induce protein expression and grown for 16 h at 30°C and 180 rpm. 1 mM of the unnatural amino acid *p*CMF and 0.1 mM δ‐aminolevulinic acid were added after induction.

After different protein expressions, cells were harvested by centrifugation (5000*g*, 4°C, 15 min) and resuspended in a lysis buffer that contained 20 mM Tris–HCl pH 8.0, 800 mM NaCl, 10 mM imidazole (for SET/TAF‐Iβ expressing cells) (González‐Arzola et al. 2015) or 10 mM Tricine‐NaOH pH 8.5 (for C*c* species expressing cells) (Gomila et al. 2022), supplemented in both cases with 0.02 mg mL^−1^ DNase I, 0.2 mg mL^−1^ lysozyme, 0.01% (w/v) phenylmethylsulfonyl fluoride (PMSF), and cOmplete™ Protease Inhibitor Cocktail (Roche). Cells were ruptured by sonication (cycles of 30 s at 35% of amplitude, 60 s of rest, 5 min total time, on ice) and cell debris were discarded by centrifugation (27,000*g*, 4°C, 60 min).

SET/TAF‐Iβ was purified by nickel affinity chromatography (González‐Arzola et al. 2015). Protein fractions were checked by SDS‐PAGE, dialyzed against 10 mM sodium phosphate, pH 7.4, and concentrated using Amicon™ Ultra‐30 Centrifugal Filter Units (Millipore). SET/TAF‐Iβ protein concentration was determined by the Bradford method (Bradford 1976), using the DC Protein Assay (Bio‐Rad Laboratories). SET/TAF‐Iβ protein concentration is expressed as the functional homodimer form.

C*c* purification was performed as previously reported through cation exchange chromatography (Gomila et al. 2022). Purity was tested by UV/Vis‐spectrophotometry and by SDS‐PAGE (Gomila et al. 2022; Navarro et al. 1995b). For all the C*c* samples used, the ratio between absorbance at 280 nm (total protein) and that at 550 nm was lower than 1.1. Fractions containing pure C*c* were dialyzed against 10 mM sodium phosphate, pH 7.4, and concentrated in a Amicon™ Ultra‐10 Centrifugal Filter Unit (Millipore). Protein concentration was determined by UV/Vis spectrophotometry, using an extinction coefficient of 28.92 mM^−1^ cm^−1^ for C*c* WT and mutant species.

### 4.4 UV/visible absorption spectroscopy

The reversibility of the heme‐iron redox state within the different C*c* species was monitored by UV/visible spectroscopy (Díaz et al. 1994; Navarro et al. 1995a). Absorption spectra of 5 μM C*c* reduced—in the presence of 50 equivalents of sodium ascorbate (Medina et al. 1992, 1993)—or oxidized—with 10 equivalents of potassium ferricyanide—species in 10 mM sodium phosphate buffer (pH 7.4) were recorded in the 350–600 nm range at RT, using a Jasco™ V‐650 spectrophotometer in a 1‐mL quartz cuvette with a path length of 10 mm.

The Fe^3+^‐Met80(S_δ_) coordination bond was monitored as previously described (Guerra‐Castellano et al. 2015; Márquez et al. 2021). UV/visible absorption spectra were recorded in the 600–750 nm range at 25°C, using a Jasco™ V‐650 spectrophotometer in a 1‐mL quartz cuvette with a path length of 10 mm. Samples contained 0.2 mM oxidized C*c* in 10 mM sodium phosphate (pH 5.8), supplemented with 0.2 mM potassium ferricyanide. For pH titration studies, the pH of each sample was adjusted to increasing pH values (6.00, 6.96, 7.22, 8.12, 9.44) by adding aliquots of 0.1–0.5M NaOH or 0.1–0.5M HCl. To calculate the protein p*K*_a_ value, the absorbance intensity changes at 699 nm were fitted to the Henderson‐Hasselbalch equation, expressed in the following terms (Márquez et al. 2021),

(4) $$Y=\mathrm{Ab}s_{\min}+\frac{\mathrm{Ab}s_{\max}-\mathrm{Ab}s_{\min}}{1+\exp\left\{\frac{X-pK_{a}}{N_{S}}\right\}},$$

where *Y* is the experimental absorbance at 699 nm, Abs_max_ and Abs_min_ are the maximum and minimum values for absorbance at 699 nm, *X* is the pH of the protein sample, p*K*_a_ is the apparent p*K*_a_ value for alkaline transition and *N*_S_ is the slope of the sigmoid.

### 4.5 Circular dichroism

Circular dichroism (CD) spectra were recorded on a Jasco™ J‐815 spectropolarimeter, equipped with a Peltier temperature control device, in a 1‐mm quartz cuvette. CD intensities were presented in terms of molar ellipticity (*θ*_molar_), based on molar protein concentration (Kelly et al. 2005).

The secondary‐structure elements of WT and mutant C*c* were analyzed by recording each far‐UV CD spectra (185–250 nm) at 25°C. Samples contained 30 μM C*c* species in 10 mM sodium phosphate (pH 7.4), supplemented with 100 μM potassium ferricyanide to keep C*c* oxidized.

The coordination state of the heme iron atom in the recombinant C*c* species was inferred by monitoring the Fe‐Met80(S_δ_) bond, the sixth axial ligand of the heme group, by recording the visible CD (350–450 nm) at 25°C in a 10‐mm quartz cuvette. Samples contained 30 μM C*c* in 10 mM sodium phosphate (pH 7.4), supplemented with 100 μM potassium ferricyanide to keep C*c* oxidized.

### 4.6 Electrochemistry

Cyclic voltammetry experiments were performed with an AUTOLAB PGTSTAT‐30 (Eco Chemie B. V.) in a three‐electrode undivided glass cell with a gas inlet, in which a Ag/AgCl/NaCl(saturated) electrode and a Pt bar act as reference and counter electrodes. Measured potential against the Ag/AgCl/NaCl(saturated) reference electrode were corrected to the normal hydrogen electrode (NHE) scale by the addition of +0.192 V to the measured potential values. The working electrode employed was a polycrystalline gold disk with a geometric area of 0.0314 cm^2^. To control the experimental temperature, the cell was also thermostated with a water jacket. The reference electrode was connected to the cell solution via a salt bridge and kept at room temperature (25 ± 2°C) in a non‐isothermal configuration. The measurements were carried out in a working solution containing 20 mM sodium phosphate buffer at pH 7.0 and under argon atmosphere. Prior to measurements, the gold surface of working electrode was successively polished with 0.3 and 0.05 μm alumina and rinse with Millipore water. The electrode was subsequently sonicated in absolute ethanol for 10 min to remove residual alumina and dried with a stream of pure nitrogen. Then, the gold surface was chemically cleaned with “*piranha*” solution composed by a 7:3 mixture of concentrated H_2_SO_4_ and 30%v/v H_2_O_2_, rinsed vigorously with Millipore water and, functionalized with a pure SAM of MOA by the immersion of the polycrystalline electrode into an ethanolic solution of 1 mM 8‐mercaptooctanoic acid (Sigma‐Aldrich) for 45 min at room temperature. The protein immobilization was carried out by a deposition of a 15 μL drop of a 25 μM C*c* and 10 mM sodium phosphate buffer at pH 7.0 solution onto the SAM‐modified electrode, for 1 h at 4°C. Cyclic voltammetry was performed at scan rates between 0.02 and 200 V s^−1^, with positive feedback for ohmic drop compensation for scan rates greater than 1 V s^−1^. Temperature variable cyclic voltammograms were acquired at equispaced temperatures between 0 and 40°C, being the upper limit dictated by the onset of protein desorption.

### 4.7 Isothermal titration calorimetry

ITC experiments were performed using a Nano ITC Low Volume instrument (TA Instruments) at the Biomolecular Interaction Platform facility (BIP‐cicCartuja, Spain). The reference cell was filled with distilled water. The experiments consisted of 17 successive 2.91‐μL injections of a 300 μM reduced‐C*c* solution into the sample cell, which contained 30 μM SET/TAF‐Iβ in the same buffer, at 25°C. C*c* and SET/TAF‐Iβ samples were dialyzed against 10 mM sodium phosphate pH 7.4 and degassed prior to the titration. Homogeneous mixing in the cell was achieved by maintaining the stirring speed at 300 rpm. Time intervals of 180 s were set to ensure the thermal power signal returned to the baseline prior to the next injection. Titration data (i.e., heat‐per‐injection normalized per mol of injectant versus molar ratio) were analyzed considering a stoichiometry C*c*:SET/TAF‐Iβ of 1:2 using NanoAnalyze software (TA Instruments) and Origin 7.0 (OriginLab) with models of two cooperative binding sites or independent binding sites. In the case of two cooperative binding sites, the parameter *n* accounted for the percentage of active protein (SET/TAF‐Iβ) in the calorimetric cell, because the 1:2 stoichiometry is implicitly established in the model. However, in the case of identical an independent binding sites, the parameter *n* accounted for the stoichiometry and the percentage of active protein (SET/TAF‐Iβ) in the calorimetric cell in a global manner.

### 4.8 Nuclear magnetic resonance‐based assays of histone binding competition

1D ^1^H NMR spectra were recorded to monitor the Met80‐εCH_3_ signal of the reduced C*c* (13 μM) as a measure of the bound protein fraction (Banci et al. 1998; Díaz‐Moreno et al. 2005b; Díaz‐Moreno et al. 2005c). First, C*c* samples were titrated with 1.75 and 3.5 μM SET/TAF‐Iβ to determine the minimum concentration at which the Met80‐εCH_3_ signal was fully broaden. A concentration of 3.5 μM SET/TAF‐Iβ was chosen for competition experiments, since it was the minimal concentration needed for the total broadening of the Met80‐εCH_3_ signal of WT and Y48*p*CMF C*c*. 1D ^1^H NMR measurements of reduced C*c* (13 μM) in presence of 3.5 μM BSA were registered as a negative control of the interaction.

To perform the histone competition assay, SET/TAF‐Iβ and C*c* concentrations were kept constant at 3.5 and 13 μM, respectively, and increasing amounts (5–40 μg) of a calf thymus histone mix (Roche, ref. 10223565001) were added.

NMR measurements of reduced C*c* were registered in 10 mM sodium phosphate buffer, pH 7.4, in the presence of 5 equivalents of sodium ascorbate (65 μM) to maintain the reduced state of C*c* (Díaz‐Moreno et al. 2005a). Spectra were acquired on a Bruker Avance 500 MHz equipped with a cryoprobe at 298 K. Water signal was suppressed according to the excitation sculpting solvent suppression method (Hwang and Shaka 1995). To adjust the lock signal, 10% D_2_O was added. 200 μL samples were prepared in 3‐mm NMR tubes.

### 4.9 Cell cultures

WT or C*c* ^−/−^ mouse fibroblast lines were kindly provided by Prof. Enriquez (CNIC, Spain) and developed by Moraes' group (Vempati et al. 2007). They were cultured in high‐glucose (4.5 g L^−1^) Dulbecco's modified Eagle's medium (DMEM, Sigma‐Aldrich), supplemented with 10% heat‐inactivated fetal bovine serum (FBS) (Sigma‐Aldrich), 2 mM L‐glutamine (Sigma‐Aldrich), 100 U/mL streptomycin (Sigma‐Aldrich), 100 μg mL^−1^ penicillin (Sigma‐Aldrich) and 1 mM sodium pyruvate (Sigma‐Aldrich). For C*c* ^−/−^ MEF, medium was also supplemented with 0.05 mg mL^−1^ uridine (Sigma‐Aldrich). Cells were grown at 37°C in a humidified atmosphere of 5% CO_2_. Cells were routinely screened for mycoplasma contamination by PCR, using the following primers: 5′‐GGCGAATGGGTGAGTAACACG‐3′ and 5′‐CGGATAACGCTTGCGACCTAT‐3′.

For fluorescence microscopy experiments, C*c* ^−/−^ MEF were grown to ca. 80% confluence in 10–24‐well plates with poly‐L‐lysine pre‐coated 20‐mm glass coverslips. Cells were then transfected with the indicated DNA construct using Lipofectamine 3000 (Invitrogen) according to the manufacturer's instructions. Briefly, 0.5 μg of DNA and 1 μL of P3000 reagent were diluted in 25 μL of Opti‐MEM medium (Invitrogen), and 1 μL of Lipofectamine was diluted in 25 μL of Opti‐MEM medium. The first solution was added to the second and incubated for 15 min at RT. Finally, the mixture was added to the cells. For protein expression, cells were incubated at 37°C for 24 h.

### 4.10 Immunofluorescence assays

Cells were fixated with 4% paraformaldehyde, washed with PBS and permeabilized with 0.1% Triton X‐100/PBS for 10 min at RT. Afterwards, cells were washed twice with PBS and incubated with blocking buffer (5% BSA/PBS) for 30 min at room temperature (RT).

For the detection of C*c* and γ‐H2AX, cells were then incubated with rabbit anti‐C*c* serum, obtained from male rabbits immunized with 20 μg mL^−1^ recombinant C*c* in 0.85% NaCl and with mouse anti‐phospho‐histone H2AX, clone JBW301 (Ser139, Sigma‐Aldrich, 05–636), both at a 1:200 dilution in blocking buffer overnight at 4°C, washed three times in PBS for 5 min and probed with goat anti‐rabbit IgG‐FITC (Sigma‐Aldrich, F9887) and goat anti‐mouse IgG Alexa Fluor 568 (Abcam, ab175473) antibodies, both 1:400 diluted in blocking buffer for 1 h at RT. Nuclei were stained by incubation with 200 μg mL^−1^ Hoechst dye (Sigma‐Aldrich) in PBS for 15 min at RT. Slides were mounted using n‐propyl gallate mounting medium and sealed with nail polish.

Samples were imaged using a Leica DM6000B (Leica Microsystems) fluorescence microscope with a 63× oil objective and an ORCA‐ER camera (Hamamatsu). Differential interference contrast (DIC) and fluorescence filters were used: a FITCL5 filter (excitation bandpass, 480/40; emission bandpass, 527/30), a Tx2 filter (excitation, 560/40 nm; emission, 645/75 nm), and an A4 filter (excitation, 360/40 nm; emission, 470/40 nm) to visualize FITC, γ‐H2AX or PLA spots, and Hoechst, respectively. Images were processed and analyzed using the LAS X (Leica Microsystems) and the FIJI (ImageJ; Schindelin et al. 2012) software packages.

### 4.11 *In situ* proximity ligation assay

For the proximity ligation assay (PLA), following fixation, permeabilization, and blocking as described above, cells were incubated with mouse anti‐C*c*, clone 6H2.B4 (BD Pharmingen, 556432) at a 1:200 dilution in blocking buffer for 1 h, in order to monitor the transfection and C*c* expression in C*c* ^−/−^ MEFs. Coverslips were then washed twice with PBS for 5 min and probed with a goat anti‐mouse IgG‐FITC antibody (Sigma‐Aldrich, F0257) 1:100 diluted in blocking buffer, for 1 h at RT.

After the cells were immunostained, PLA was performed to detect the interaction between Myc‐tagged C*c* species and SET/TAF‐Iβ. Mouse anti‐Myc tag, clone 4A6 (Sigma‐Aldrich, 05‐724) and rabbit anti‐SET (SinoBiological Inc., 101069‐T46) at 1:200 dilutions were added to the slides and incubated for 1 h at RT. As a negative control, the anti‐Myc antibody was used together with an irrelevant rabbit primary antibody. PLA was then performed using DuoLink™. *In situ* PLA donkey anti‐rabbit PLUS and anti‐mouse MINUS probes (Sigma‐Aldrich, DUO92002 and DUO92004) and Detection Reagents Red (Sigma‐Aldrich, DUO92008) according to the manufacturer's instructions. Nuclei staining, slide mounting, and sample imaging and analysis were performed as described for immunofluorescence assays. Nuclear PLA spots were quantified in approximately 30 transfected cells per condition from 3 biological independent replicates, following the protocol to visualize and analyze the images described by Prado Martins et al. (Prado Martins et al. 2018). A Shapiro–Wilk normality test was conducted for each condition. Due to the lack of normality, a Mann–Whitney test was used to assess mean differences among conditions.

## AUTHOR CONTRIBUTIONS

**Joaquín Tamargo‐Azpilicueta:** Investigation; methodology; formal analysis; visualization; writing – original draft; writing – review and editing; conceptualization. **Miguel Á. Casado‐Combreras:** Writing – review and editing; investigation; visualization; formal analysis; methodology. **Rafael L. Giner‐Arroyo:** Investigation; formal analysis; visualization; writing – review and editing; methodology. **Adrián Velázquez‐Campoy:** Formal analysis; writing – review and editing; visualization; investigation; methodology. **Inmaculada Márquez:** Investigation; methodology; formal analysis. **José L. Olloqui‐Sariego:** Investigation; methodology; formal analysis. **Miguel A. De la Rosa:** Supervision; funding acquisition; conceptualization; writing – review and editing; resources; investigation; methodology. **Irene Diaz‐Moreno:** Supervision; funding acquisition; resources; conceptualization; project administration; investigation; writing – review and editing; data curation; writing – original draft; methodology.

## Supporting information


**Data S1.** Supporting Information.
