# Supplementary material for: Phosphorylation of cytochrome c at tyrosine 48 finely regulates its binding to the histone chaperone SET/TAF‐Iβ in the nucleus
Source: Protein Sci. 2024 Nov 16;33(12):e5213. doi: 10.1002/pro.5213 (PMC11568366; doi:10.1002/pro.5213)
Supplement: Supplementary file 1 — Data S1. Supporting Information. [file PRO-33-e5213-s001.docx]

**Phosphorylation of cytochrome *c* at tyrosine 48 finely regulates its binding to the histone chaperone SET/TAF-Iβ in the nucleus**

Joaquín Tamargo-Azpilicueta^1^, Miguel A. Casado-Combreras^1^, Rafael L. Giner-Arroyo^1^, Adrián Velázquez-Campoy^2,3,4,5^, Inmaculada Márquez^6^, José L. Olloqui-Sariego^6^,
Miguel A De la Rosa^1^, Irene Diaz-Moreno^1,*^

^1^Institute for Chemical Research (IIQ), Scientific Research Center “Isla de la Cartuja” (cicCartuja), University of Seville – CSIC, Seville, Spain.

^2^Institute for Biocomputation and Physic of Complex Systems (BIFI), Joint Unit GBsC-CSIC-BIFI, University of Zaragoza, Zaragoza, Spain.

^3^Departament of Biochemistry and Molecular and Cellular Biology, University of Zaragoza, Zaragoza, Spain.

^4^Institute for Health Research Aragón (IIS Aragon), Zaragoza, Spain.

^5^Centre for Biomedical Research Network of Hepatic and Digestive Diseases (CIBERehd), Madrid, Spain.

^6^Department of Physical-Chemistry, University of Seville, Seville, Spain.

**^*^Corresponding author:**

Irene Díaz-Moreno

Mailing address: C. Américo Vespucio, 49 (cicCartuja). 41092, Seville (Spain).

Telephone number: +34 954489513

Email address: [idiazmoreno@us.es](mailto:idiazmoreno@us.es)


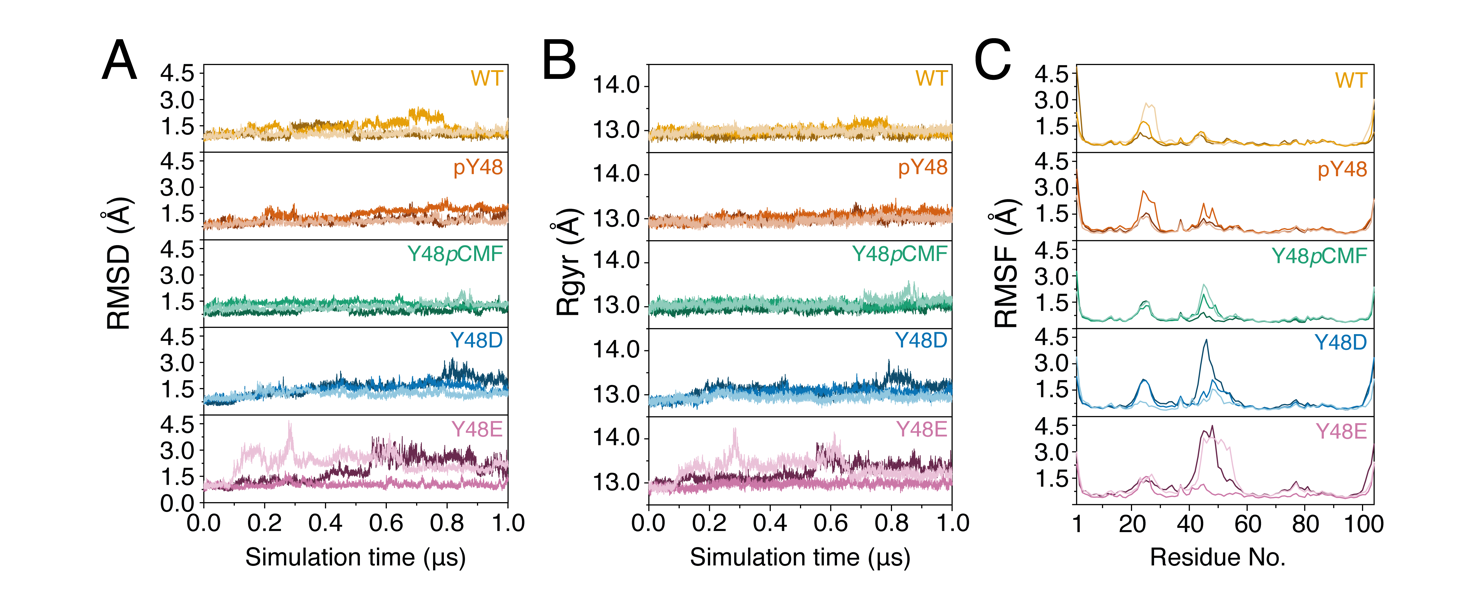


**Figure S1. Statistical analysis of molecular dynamics simulations trajectories.** (A) Backbone atomic root-mean-square deviations (RMSD), (B) radius of gyration (*R*_gyr_,) and (C) root-mean-square fluctuations (RMSF) of reduced unmodified and phosphorylated WT or Y48*p*CMF, Y48D or Y48E phosphomimetic C*c* species.

**
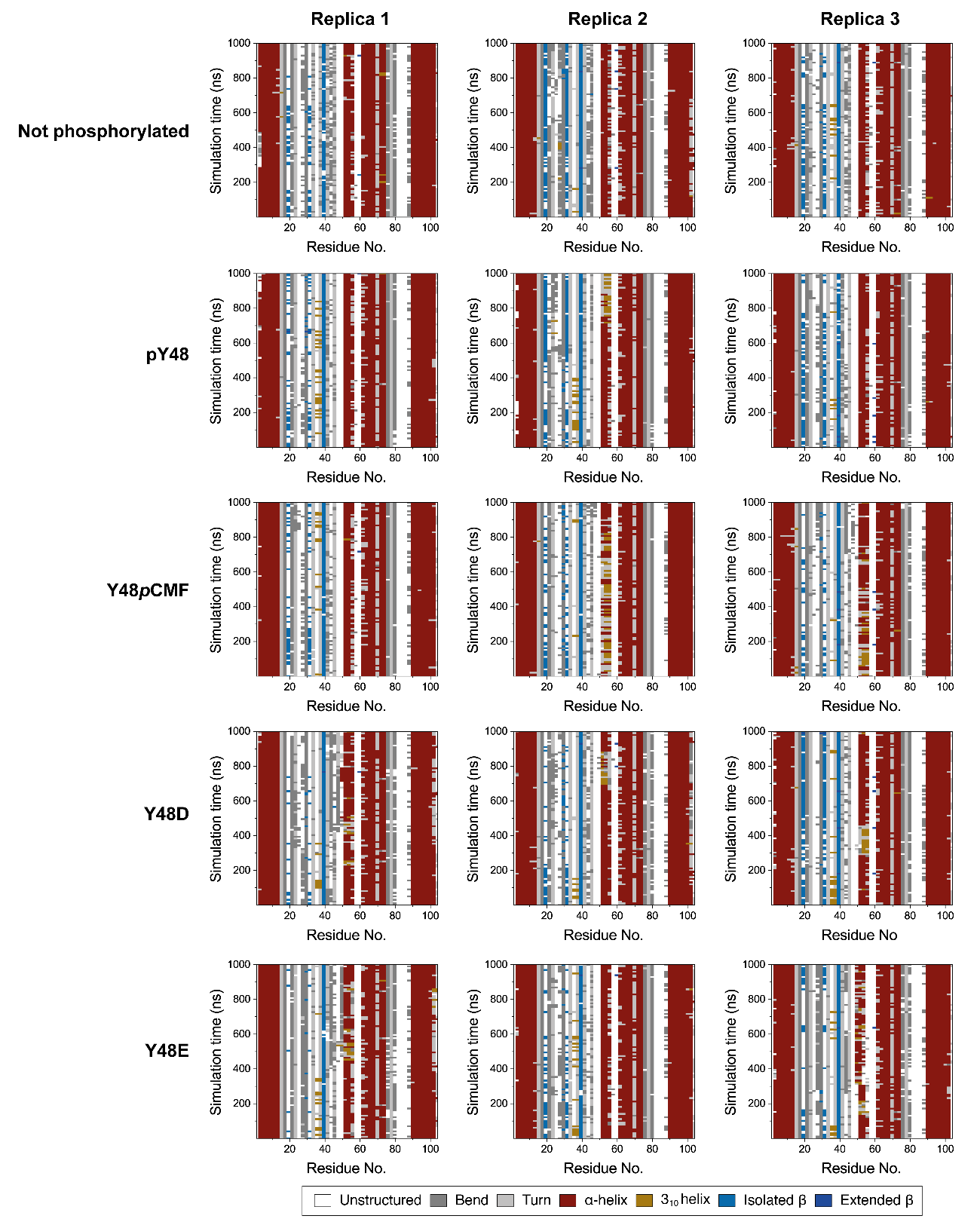
**

**Figure S2. Secondary structure analyses of reduced WT and mutant cytochrome *c* species.** Plots of definition secondary structure of protein (DSSP) for unmodified WT**,** phosphorylated (pY48) and phosphomimetic Y48*p*CMF, Y48D and Y48E C*c* variants along 1-μs MD simulations.


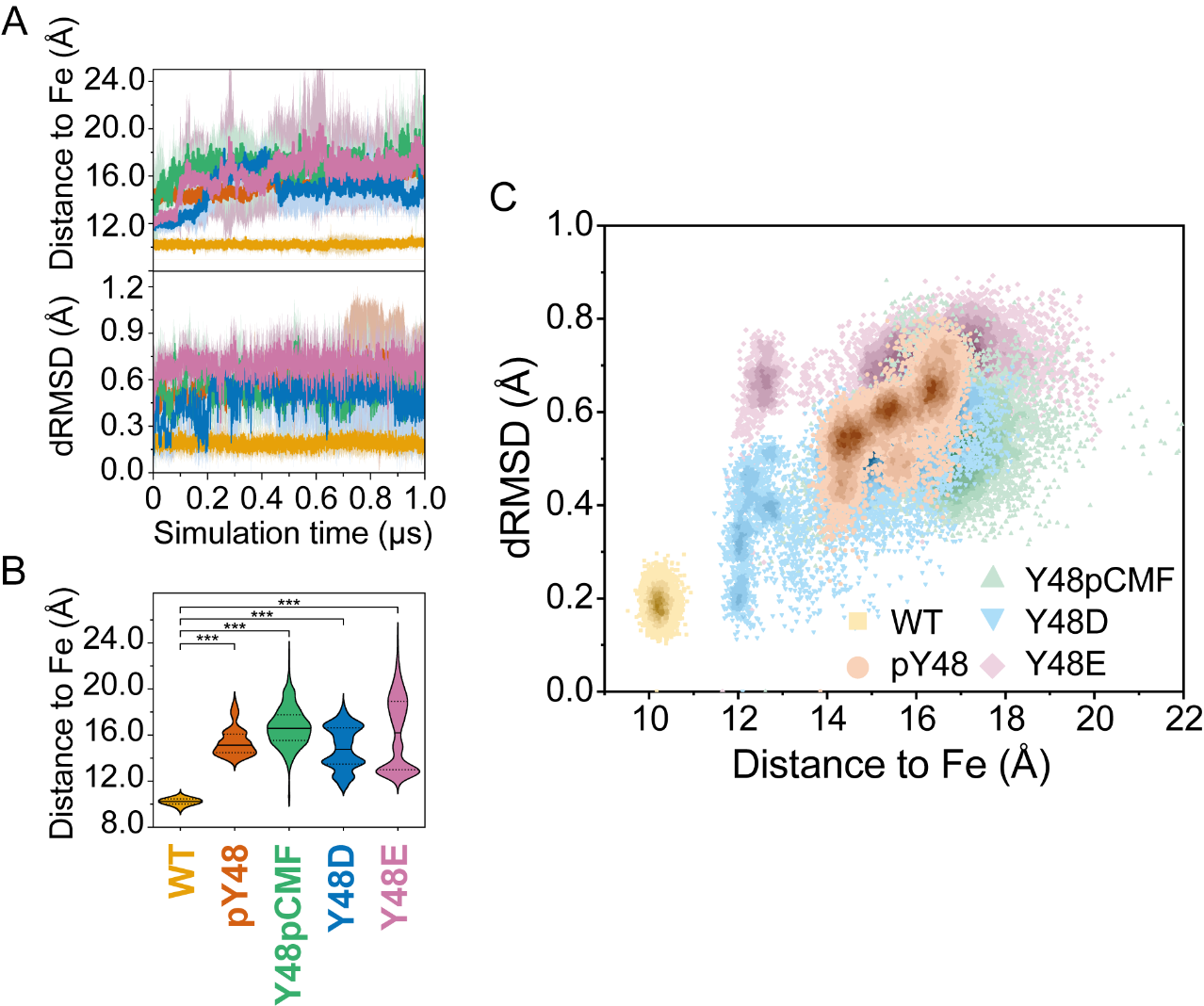


**Figure S3. Intramolecular distances progression during molecular dynamics simulations of reduced WT and phosphomimetic cytochrome *c*. (A)** Distance progression of the centers of mass of the residue in position 48 to heme Fe during the trajectory (*upper panel*) and distance RMSD of the atoms of residue in position 48 to the rest of atoms in the protein using the structure at t = 0 ns as a reference (*lower panel*). Average values are represented as thick lines, and standard deviation (± standard error for n = 3 replicas) for each replica is represented by a shaded envelope. **(B)** Violin plot of the distances between position 48 centers of mass and the heme Fe (10000 points per trajectory were analyzed; significance level: ∗∗∗*p* < 0.001). **(C)** Scatter plot (n = 3, 10000 points per replica) of the dRMSD and the position 48-heme Fe distance values described in panel A. Darker color indicates higher density of points.


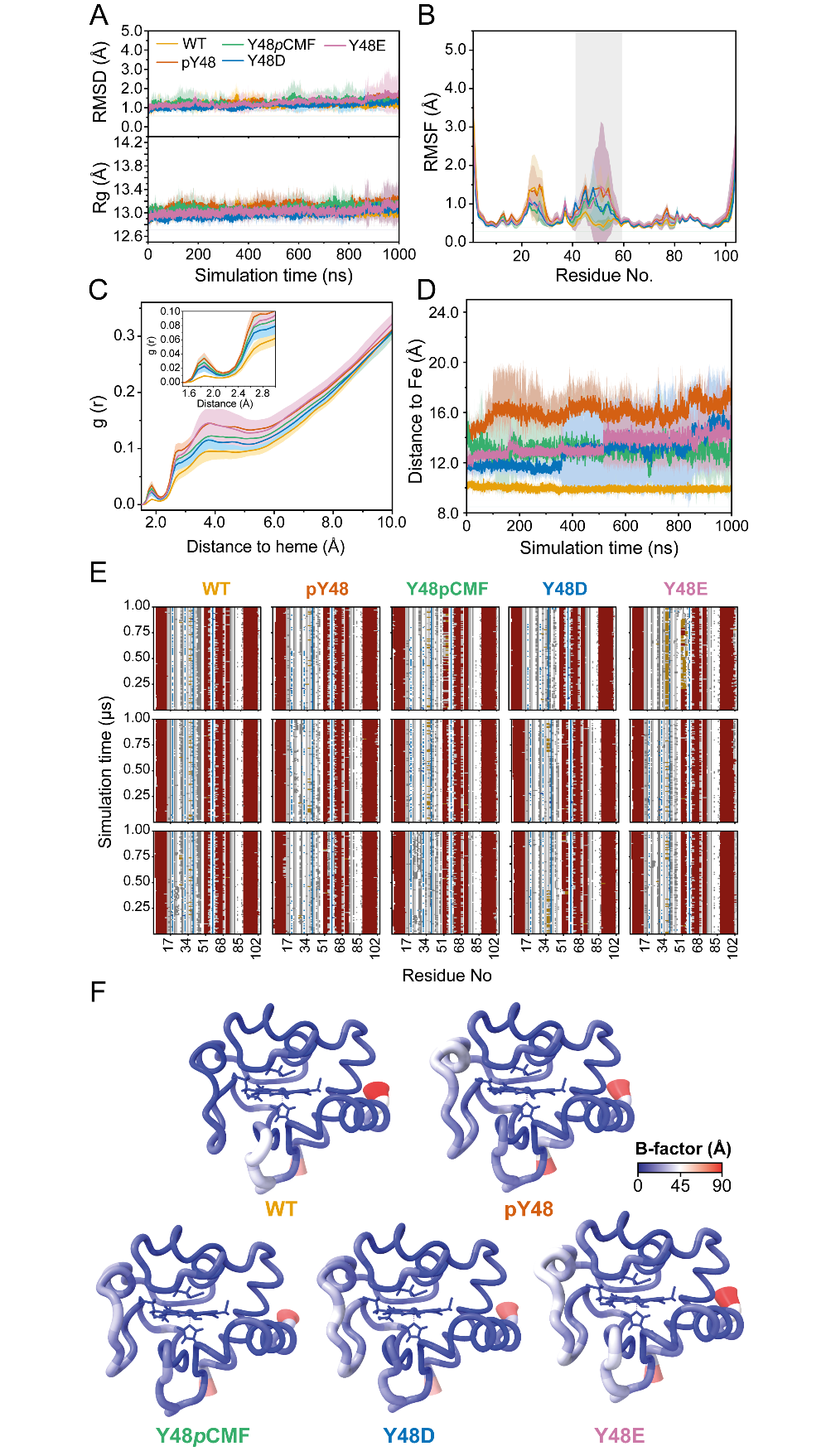


**Figure S4. Molecular dynamics simulations of oxidized WT, Tyr48-phosphorylated and phosphomimetic cytochrome *c* species. (A)** Average backbone atomic root-mean-square deviations (RMSD, *bottom panel*) and radius of gyration (*Rg*, *top panel*) for the three MD simulation runs. **(B)** Average atomic root-mean-square fluctuations (RMSF) per C*c* residue. Region corresponding to foldon V (residues 40-57), which contains Tyr48, is shadowed in gray. **(C)** Radial distribution function, $g(r)$, of water molecules around the C*c* porphyrin ring. A spherical region of 10 Å radius around the heme group was analyzed. **(D)** Distance progression of the centers of mass of the residue in position 48 to heme Fe during the trajectory. Average values are represented as thick lines, and standard deviation (± standard error for n = 3 replicas) for each replica is represented by a shaded envelope.  **(E)** Plots of definition secondary structure of protein (DSSP) for unmodified WT**,** phosphorylated (pY48) and phosphomimetic Y48*p*CMF, Y48D and Y48E C*c* variants along 1-μs MD simulations. **(F)** Ribbon structure of the average molecular dynamic trajectories of WT, pY48 or phosphomimetic C*c* variants depicted according to its dynamical properties (i.e., the B-factor) from blue and thin (low flexibility) to red and thicker (high flexibility).

**
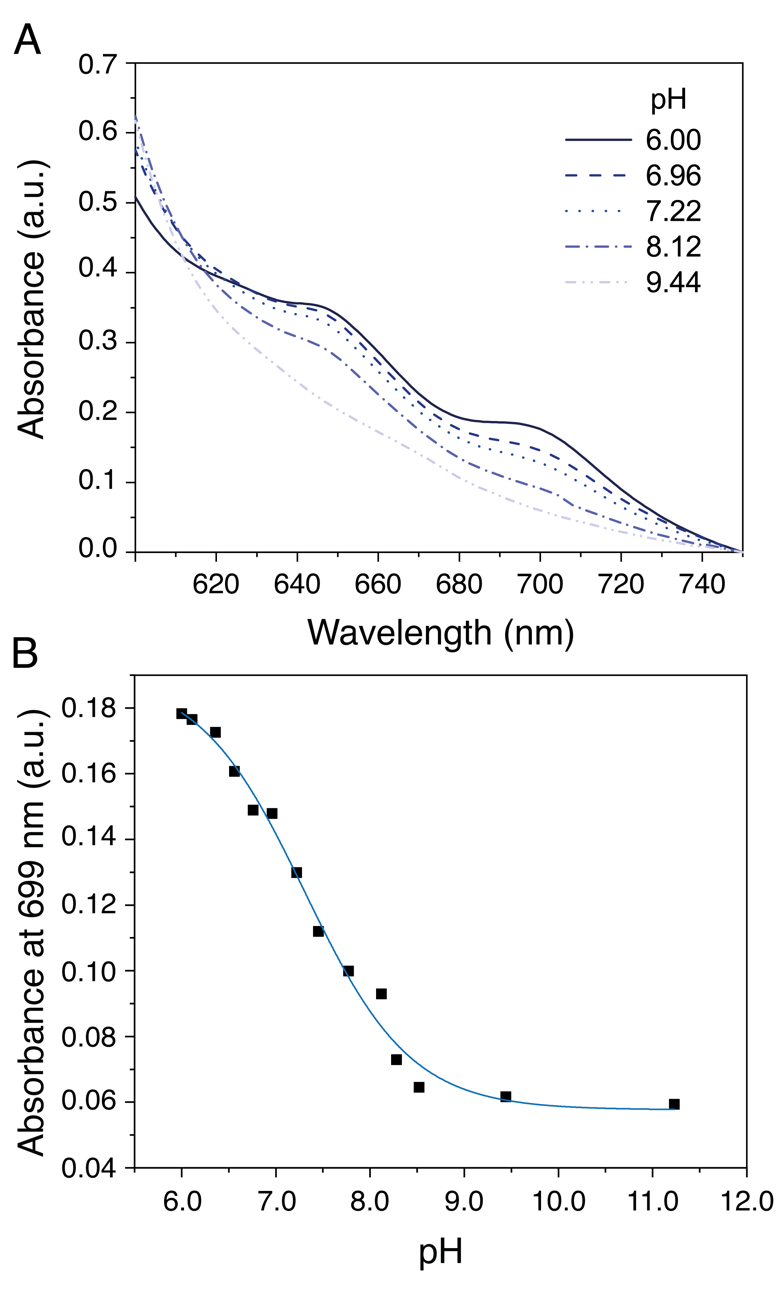
**

**Figure S5. Alkaline transition for the oxidized Y48D cytochrome *c* mutant. (A)** UV/visible absorption spectra of Y48D C*c* recorded at different pH values. **(B)** pH-dependence of absorbance intensity at 699 nm, as fitted to the Henderson-Hasselbalch function (continuous line).

**
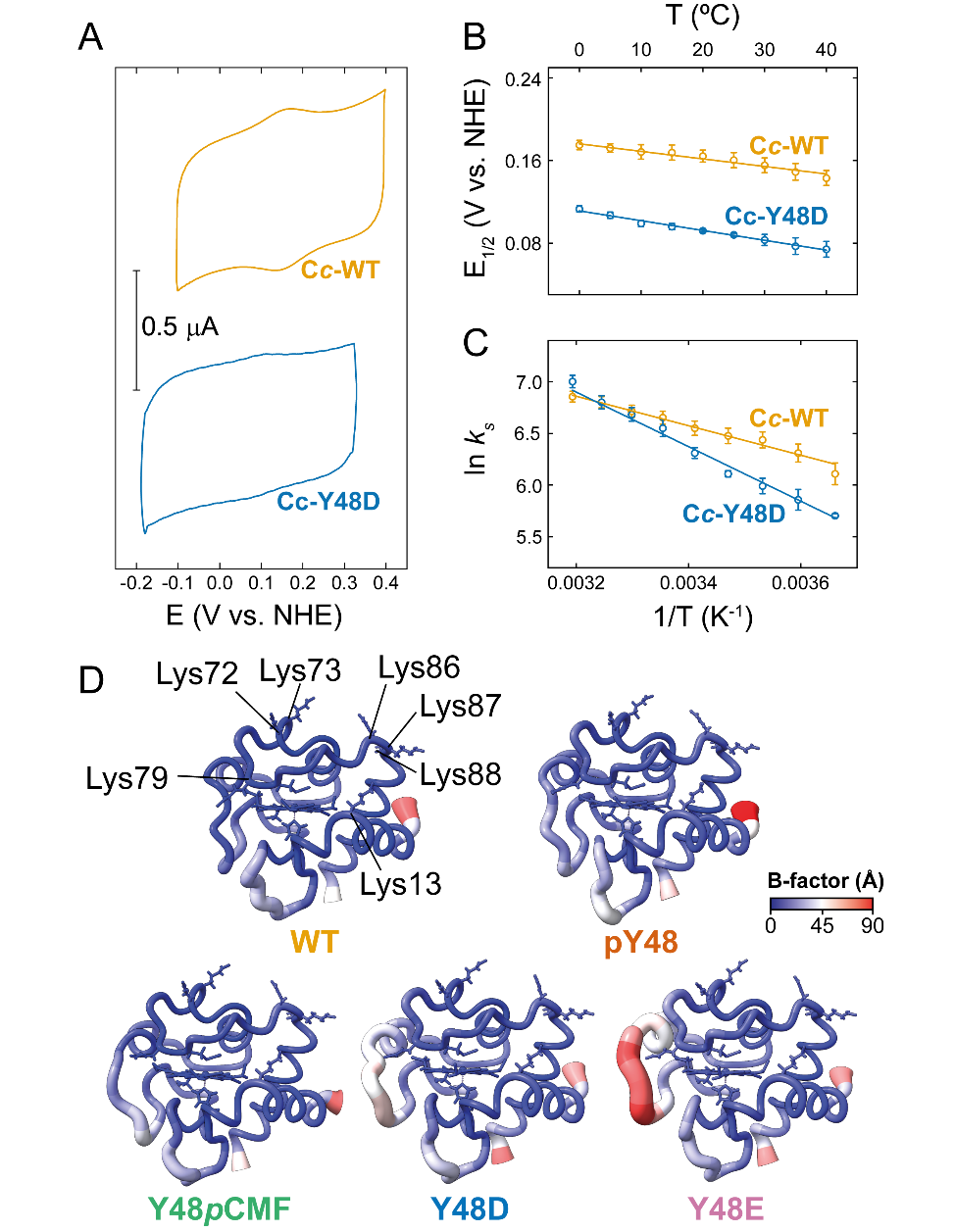
**

**Figure S6. Electrochemical characterization of WT and Y48D cytochrome *c* species.** (**A**) Raw cyclic voltammograms recorded at 0.5 V s^‑1^ and 25 ºC. (**B**) Temperature-dependence of the midpoint potential value (*E_1/2_*), (**C**) Arrhenius plots of the standard electron transfer rate constant (*k_s_*) of C*c* immobilized onto a gold electrode modified with 8-mercaptooctanoic acid SAMs. Solid lines are the best linear least-squares data fits. Buffer was 20 mM sodium phosphate, pH 7. (**D**) Ribbon structure of the average molecular dynamic trajectories of WT, pY48 or phosphomimetic C*c* variants illustrated according to its dynamical properties (i.e., the B-factor) from blue and thinner (low flexibility) to red and wider (high flexibility). Lysine residues involved in the interaction with the SAM surface are highlighted in the WT structure and depicted in all the structures.

**
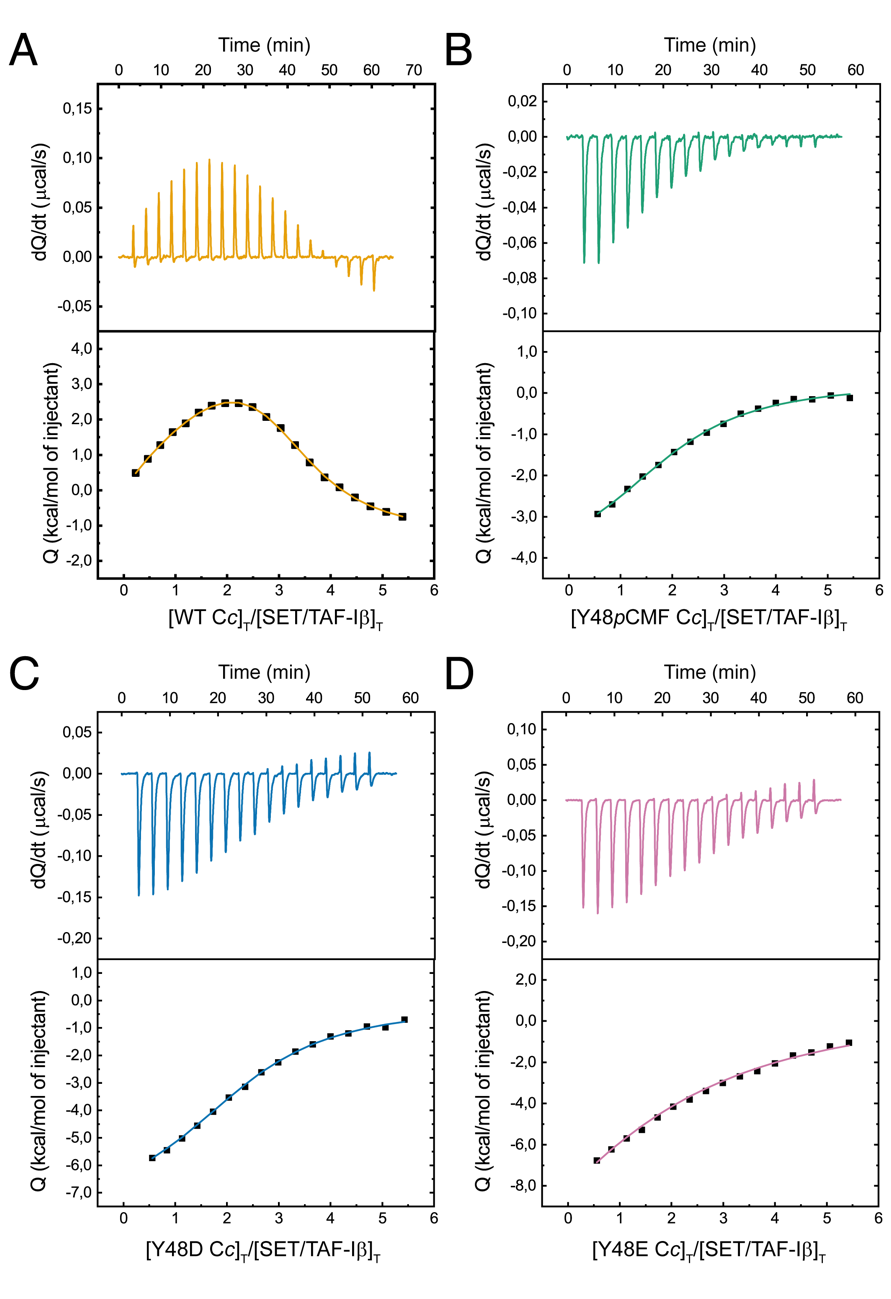
**

**Figure S7. ITC binding assays between SET/TAF-Iβ and cytochrome *c* species.** Thermograms (*upper panels*) and binding isotherms (*lower panels*) of reduced WT (**A**), Y48*p*CMF (**B**), Y48D (**C**) and Y48E (**D**) C*c* species upon binding to SET/TAF-Iβ.

**
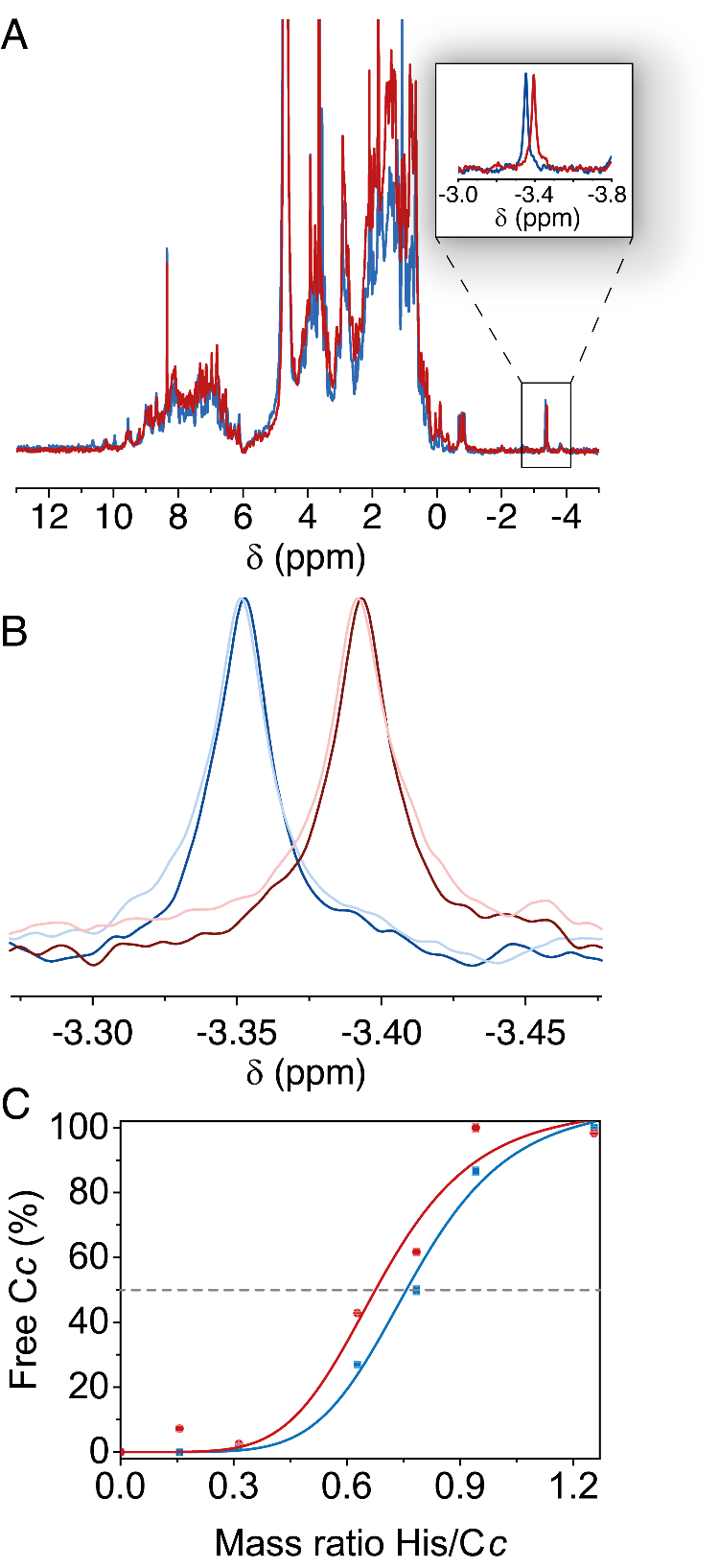
**

**Figure S8. NMR** **analyses on the WT and Y48D cytochrome *c* species.** (**A**) 1D ^1^H NMR spectra of reduced WT (*blue*) and Y48*p*CMF (*red*) C*c*. Inset: Met80-ε-CH_3_ signal of free reduced, WT or Y48*p*CMF C*c*. (**B**) Detailed view of the 1D ^1^H NMR spectra monitoring WT (*blue*) and Y48*p*CMF (*red*) C*c* Met80-εCH_3_ signal in free-state (*dark lines*) and in the presence of 3.5 μM BSA (*light lines*). **(C)** Free C*c* percentage was estimated from the Met80-εCH_3_ maximum intensity signal, normalized to *B*_max_ and plotted against the histone:C*c* (His/C*c*) mass ratio included in Figure 3. The Hill equation was fitted to the titration data points to estimate the half maximal inhibition concentration (EC50) for WT (0.77 ± 0.026, adj. R^2^ = 0.990, blue line) and Y48*p*CMF C*c* (0.69 ± 0.046, adj. R^2^ = 0.964, red line). Error bars represent the fit standardized residual.

**
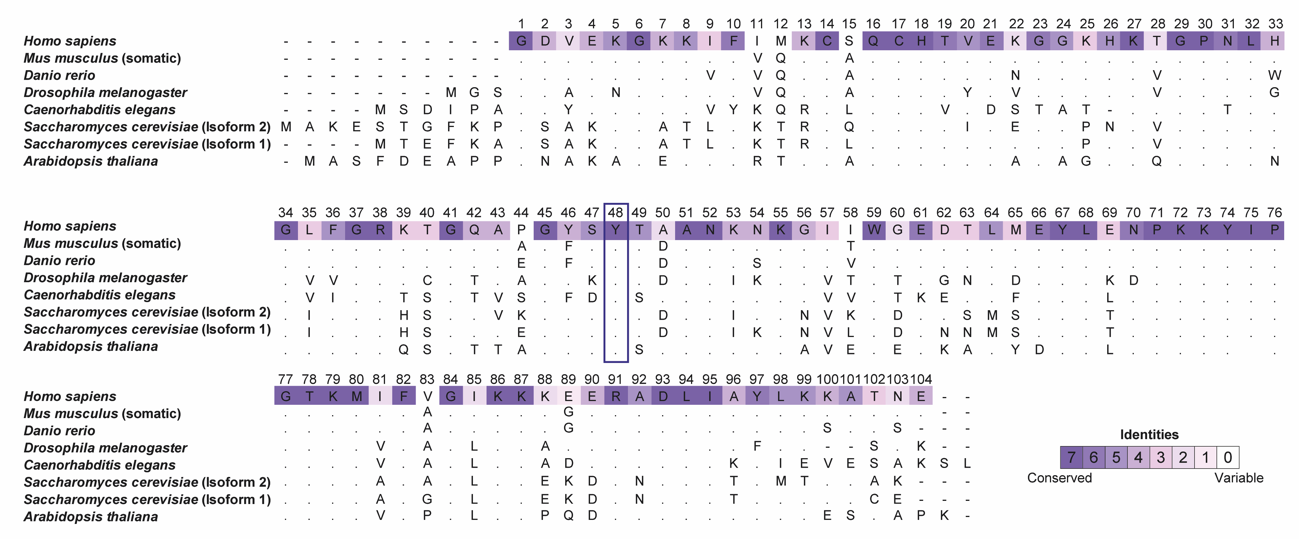
**

**Figure S9. Cytochrome *c* Tyr48 is phylogenetically conserved** **in eukaryotes**. Residues in *Homo sapiens* sequence was colored according to the identities across all analyzed species: metazoans (*Homo sapiens, Mus musculus, Danio rerio, Drosophila melanogaster, Caenorhabditis elegans*), fungi (*Saccharomyces cerevisiae*) and plants (*Arabidopsis thaliana*).

**Table S1. Secondary structure element distribution in the oxidized WT and mutant cytochrome *c* species.** Fractional contents in secondary structure elements of the WT, Y48D, Y48E and Y48*p*CMF C*c* species were inferred from far-UV CD spectra using the DichroWeb software (Miles et al., 2022) and SP175 reference database (Lees et al., 2006).

| **C*c* species** | **Regular  α-helix** | **Distorted  α-helix** | **Regular  β-strand** | **Distorted  β-strand** | **Turns** | **Random  coil** |
| --- | --- | --- | --- | --- | --- | --- |
| **WT** | 0.562 | 0.230 | 0.000 | 0.001 | 0.098 | 0.153 |
| **Y48D** | 0.514 | 0.224 | 0.004 | 0.003 | 0.101 | 0.195 |
| **Y48E** | 0.511 | 0.220 | 0.003 | 0.002 | 0.100 | 0.188 |
| **Y48*p*CMF** | 0.521 | 0.225 | 0.002 | 0.001 | 0.100 | 0.192 |

**Table S2. Soret band maximum values for the WT and phosphomimetic (Y48D, Y48E, Y48*p*CMF) C*c* mutants.**

| **Species** | **Fe(II)-C*c***  (nm) | **Fe(III)-C*c***  (nm) |
| --- | --- | --- |
| **WT** | 415 | 409 |
| **Y48D** | 414 | 406 |
| **Y48E** | 415 | 406 |
| **Y48*p*CMF** | 413 | 406 |

**Table S3. Midpoint redox potential, thermodynamic and kinetic parameters for WT and Y48D cytochrome *c*.** Midpoint redox potential value (*E_1/2_*), reduction entropy (Δ*S^0^_rc_*), reduction enthalpy (Δ*H^0^_rc_*), electron transfer rate constant (*k_s_*), pre-exponential factors (*A*) and activation enthalpy (Δ*H^#^_ET_*) are shown.

|  | $\boldsymbol{E}_{\boldsymbol{1/2}}$  (mV^a^) | $\boldsymbol{\Delta}\boldsymbol{S}_{\boldsymbol{rc}}^{\boldsymbol{0}}$(J K^-1^ mol^-1^) | $\boldsymbol{\Delta}\boldsymbol{H}_{\boldsymbol{rc}}^{\boldsymbol{0}}$  (kJ mol^-1^) | $\boldsymbol{k}_{\boldsymbol{s}}$ (s^-1^)* | **10^-6^ A** (s^-1^) | ${\boldsymbol{\Delta}\boldsymbol{H}}_{\boldsymbol{ET}}^{\mathbf{⋕}}$  (kJ mol^-1^) | **Reference** |
| --- | --- | --- | --- | --- | --- | --- | --- |
| **WT** | 160 ± 7 | -74 ± 5 | -37 ± 3 | 775 ± 25 | 0.11 ± 0.03 | 12.0 ± 0.5 | This study |
| **Y48D** | 98 ± 5 | -92 ± 7 | -36 ± 4 | 700 ± 20 | 8 ± 3 | 23.0 ± 3 | This study |
| **Y48*p*CMF** | 91 ± 5 | -115 ± 8 | -43 ± 5 | 650 ± 20 | 64 ± 4 | 28 ± 2 | Olloqui-Sariego *et al.* (2022) |
| **Y48H** | 85 ± 5 | -90 ± 5 | -34 ± 3 | 650 ± 30 | 3 ± 3 | 21 ± 2 | Olloqui-Sariego *et al.* (2022) |

*Measured at 25 °C.

- **References**

Lees, J. G., Miles, A. J., Wien, F., & Wallace, B. A. (2006). A reference database for circular dichroism spectroscopy covering fold and secondary structure space. Bioinformatics, 22:1955–1962.

Miles, A. J., Ramalli, S. G., & Wallace, B. A. (2022). DichroWeb, a website for calculating protein secondary structure from circular dichroism spectroscopic data. Protein Sci, 31:37–46.

Olloqui-Sariego, J. L., Pérez-Mejías, G., Márquez, I., Guerra-Castellano, A., Calvente, J. J., De la Rosa, M. A., Andreu, R., & Díaz-Moreno, I. (2022). Electric field-induced functional changes in electrode-immobilized mutant species of human cytochrome c. Biochim Biophys Acta 1863:148570.
